# A comparative study of temperature-dependent characteristics and non-local behavior in a submerged microstretch thermoelastic medium using two models

**DOI:** 10.1038/s41598-024-77233-9

**Published:** 2024-11-07

**Authors:** Mohamed I. A. Othman, Ebtesam E. M. Eraki, Mohamed F. Ismail

**Affiliations:** 1 Department of Mathematics, Faculty of Science, ZagazigUniversity, P.O. Box 44519, Zagazig, Egypt; 2 Faculty of Computers and Information System, Egyptian Chinese University, Cairo, Egypt

**Keywords:** Thermo-micro-stretch elastic, Normal mode method, Non-local, Frequency, Temperature dependent, Fluid, Materials science, Mathematics and computing, Physics

## Abstract

This article is concerned with a thermoelastic response in a non-local micro-stretch completely covered in the endless non-viscous fluid under temperature dependent, the medium is investigated employing the theory of Green-Naghdi (G-N III) and the model of three-phase-lag (3PHL).Basic equations are derived based on these models. The normal mode technique is employed to achieve an analysis solution to the problem. The study used a magnesium crystal element to compare non-local measurements of thermo-micro-stretch elasticity in water using the (G-N III) theory and the (3PHL) model. The non-local effect has been discovered to have a considerable impact on all physical quantities. Furthermore, comparisons are made between three different frequency values.

**Supplementary Information:**

The online version contains supplementary material available at 10.1038/s41598-024-77233-9.

## Introduction

The impact of a magnetic field on spread waves in a fiber-reinforced plate using both (G-N III) type and the (3PHL) model was focused by Othman and Said^1^. The thermo-elastic analysis at the micro and nano-scales, in which the volume impact on the conduction of heat and deformation increases and the theory of classical thermoelastic no longer controls was introduced by Yu et al^2^.. The non-local thermoelastic scenario is based on the non-local just phase-lag scenario presented by Luo et al^3^.. Singh et al^4^. conducted research on the thermoelastic scenario related to scattered waves in thermo-microstretch elastic materials under diffusion media. Marin et al. studied the impact of two temperatures on thermo-microstretch elastic. Articles^6–12^ collectively exemplify the broad spectrum of nonlocal phenomena studied in the fields of thermoelasticity. Based on the pervious papers, one infers that the constitutive relations

1 $$(\;1\; - \;\eta^{2} \,\nabla^{2} \;)\;\sigma_{ij} \; = \;[\lambda \;u_{r,r} \; + \;\lambda_{1} {\kern 1pt} \varphi^{*} \; - \;\beta_{1} {\kern 1pt} (\,T\, - \,T_{0} )\,]\;\delta_{ij} \; + \;\mu \;(u_{i,j} \; + \;u_{j,i} )\; + \;k\;(u_{j,i} {\kern 1pt} \varepsilon_{ijr} {\kern 1pt} \varphi_{r} ),$$

2 $$(\;1\; - \;\eta^{2} \,\nabla^{2} \;)\;m_{ij} \; = \;\alpha \;\varphi_{r,r} \delta_{ij} \; + \;\beta \;\varphi_{i,j} \; + \;\gamma \;\varphi_{j,i} ,$$

3 $$(\;1\; - \;\eta^{2} \,\nabla^{2} \;)\;\lambda_{k} \; = \;\alpha_{0} \;\varphi_{,k}^{*} ,\;\;\quad \quad e\; = \;u_{k,k} .$$

Kumar et al^13^.. introduced a study within the broader context of classical and non-classical thermoelastic scenarios. The research focused on the propagation of straight and round-topped waves in a micro-stretch solid bounded by liquid with varying temperatures on each side. The foregoing study utilized the generalized micro-stretch scenario to investigate this phenomenon. A model that utilized liquid-structure to explain thermo-elastic formation under laser and the spread of Leaky Lamb waves at the liquid interface was investigated by Xu et al^14^.. Depending on the last references, it can be concluded

4 $$(\lambda \; + \;\mu )\;\nabla \;(\;\nabla \;.\;{\varvec{u}}\;)\; + \;(\mu \; + \;k)\;\nabla^{2} \,{\varvec{u}}\; + \;\lambda_{1} \;\nabla \,\varphi^{*} \; - \;k\;(\,\nabla {\kern 1pt} \times \;\user2{\varphi }\,\rm{)}\; - \;\beta_{1} \;\nabla {\kern 1pt} T\; = \;\rho \,(\;1\; - \;\eta^{2} \,\nabla^{2} \;)\,\frac{{\partial^{2} {\varvec{u}}}}{{\partial \,t^{2} }},$$

this indicates that the equation of motion and notices that appearance micro-rotation vector ($\user2{\varphi }$), scalar micro-stretch function ($\varphi^{*}$), and non-local parameter ($\eta$).

5 $$(\,\alpha \, + \,\beta \, + \,\gamma \,)\,\nabla \,(\,\nabla \,.\,\user2{\varphi }\,)\; - \;\gamma \,\nabla \, \times \,(\,\nabla \, \times \,\user2{\varphi }\,)\; + \;k\,\nabla \, \times \,{\varvec{u}}\; - \;2\,k\,\user2{\varphi }\; = \;\rho {\kern 1pt} (\;1\; - \;\eta^{2} \,\nabla^{2} \;)\;J{\kern 1pt} \frac{{\partial^{2} \user2{\varphi }}}{{\partial \,t^{2} }},$$

6 $$\alpha_{0} {\kern 1pt} \nabla^{2} \;\varphi^{*} \; + \;\frac{1}{3}\;\nu {\kern 1pt} \,(\;T\; - T_{0} \;)\; - \;\frac{1}{3}\;\lambda_{1} {\kern 1pt} \varphi^{*} \; - \;\frac{1}{3}\;\lambda_{0} \;\nabla \,.\;{\varvec{u}}\; = \;\frac{3}{2}\;\rho {\kern 1pt} \;(\;1\; - \;\eta^{2} \,\nabla^{2} \;)\;J_{0} {\kern 1pt} \frac{{\partial^{2} \varphi^{*} }}{{\partial \,t^{2} }},$$

this means that the equations of micro polar and micro-stretch respectively. we observe that a non-local parameter ($\eta$) in the right side with the second derivative with respect to time.

The (3PHL) and (DPL) thermoelastic models to evaluate the spread of plane waves in an isotropic thermoelastic plate were utilized by Kumar and Chawla^15^. The fore-mentioned models are employed to examine the effect of gravity field thermoplastic isotropic solid under two-temperature fiber-reinforced by Othman et al^16^.. Within (3PHL) model, a study of the impact of gravity on a homogeneous, isotropic, thermo-micropolar elastic solid under voids was demonstrated by Alharbi^17^. Tiwari and Kumar^18^ proposed adding a non-local affect to the performance parameter of a micro-mechanical resonator. They accomplished this by using a (3PHL) scenario with a memory-dependent derivative. In the (3PHL) and (G-N III), It can be deduced that

7 $$k^{*} \nabla^{2} \,T\; + \;(k_{1}^{*} \; + \;k^{*} \tau_{\nu } ){\kern 1pt} T_{,t} \; + \;k_{1}^{*} \tau_{\theta } \nabla^{2} \,T_{,tt} \; = \;[1\; + \;\tau_{q} \frac{\partial }{\partial t}\; + \;\frac{1}{2}\;\tau_{q}^{2} \frac{{\partial^{2} }}{{\partial t^{2} }}][\rho {\kern 1pt} c_{E} T_{,tt} \; + \;\nu {\kern 1pt} T_{0} \varphi_{,tt}^{*} \; + \;\beta_{1} T_{0} e_{,tt} \;],$$

where, (G-N III) when($\tau_{\nu } \; = \;\tau_{\theta } \; = \;\tau_{q} \; = \;0,\;k^{*} \rm{ > }\;0$). This illustrates that heat conduction equation whose possess scalar micro-stretch function ($\varphi^{*}$).

The normal mode method has been employed by multiple researchers to investigate generalized thermoelastic materials, as documented in various studies^19–28^. Marin et al. studied the partition of energies for backward in time problem of the thermoelastic materials with a dipolar structure.

**Fig. 1:**
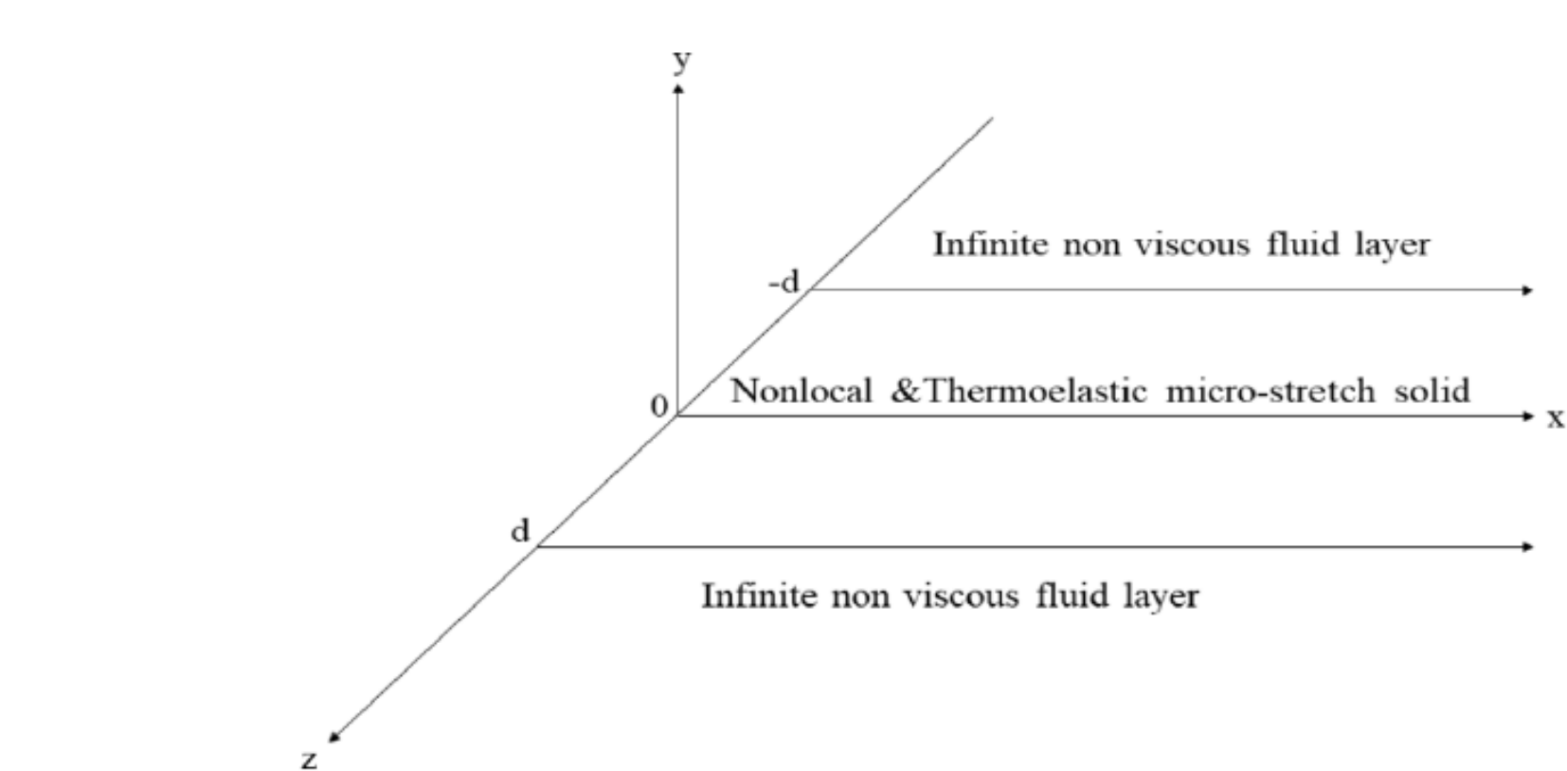
Geometry of problem.

In this study, our main focus was to demonstrate the impact of non-locality on a micro-stretch thermo-elastic material that is fully immersed in fluid with temperature dependency, as depicted in Fig. 1. We employed the (3PHL) and (G-N III) to analyze the behavior of the plate. We began by demonstrating the fundamental equations and using dimensionless. A normal mode method is used in the second step to transform the five partial differential equations into five ordinary differential equations. Following that, we created the boundary conditions at $z\; = \; \pm \;d$ to find the constant values of the solutions. At last, the calculations are implemented, discussed, and graphed.

## The description of the problem and basic equations

We aim to examine the impact of temperature-dependent thermoelastic on the various wave propagation properties. According to Deswal et al^27^., we assume that

8 $$(\,\lambda ,\,\mu ,\,\lambda_{1} ,\,\beta_{1} ,\,k,\,\alpha ,\,\beta ,\,\gamma ,\,\alpha_{0} ,\,\nu ,\,\lambda_{0} \,)\, = \,(\,\lambda^{\prime},\,\mu^{\prime},\,\lambda^{\prime}_{1} ,\,\beta^{\prime}_{1} ,\,k^{\prime},\,\alpha^{\prime},\,\beta^{\prime},\,\gamma^{\prime},\,\alpha^{\prime}_{0} ,\,\nu^{\prime},\,\lambda^{\prime}_{0} \,)\,f\;(\,T_{0} \,).$$

where $\lambda ,\,\mu ,\,\lambda_{1} ,\,\beta_{1} ,\,k,\,\alpha ,\,\beta ,\,\gamma ,\,\alpha_{0} ,\,\nu ,\,\lambda_{0}$ are constants and $f\;(\,T_{0} \,)$ is a dimensionless function of reference temperature which $f\;(\,T_{0} \,)\; = \;1\; - \;\alpha^{*} \;T_{0} .$  In case of temperature independent properties, we have $f\;(\,T_{0} \,)\; = \;1$.

From Eq. (4) - (7) for $\user2{u }\rm{(}x,\;z,\;t\rm{)}\,\rm{ = }\;u\;(u_{1} ,\;0,\;u_{3} )$ and $\user2{\varphi }\; = \;(\;0,\;\varphi_{2} ,\;0\;)$ with help Eq. (8), the governing equations can be written as

9 $$(\lambda^{\prime}\; + \;\mu^{\prime})\;e_{,x} \; + \;(\mu^{\prime}\; + \;k^{\prime})\;u_{1,ii} \; + \;\lambda^{\prime}_{1} \,\varphi_{,x}^{*} \; - \;k^{\prime}{\kern 1pt} \varphi_{2,z} \; - \;\beta^{\prime}_{1} {\kern 1pt} T_{,x} \; = \;\frac{{\rho \,(\;1\; - \;\eta^{2} \,\nabla^{2} \;)}}{{\alpha_{1} }}\,u_{1,tt} \;,\;$$

10 $$(\lambda^{\prime}\; + \;\mu^{\prime})\;e_{,z} \; + \;(\mu^{\prime}\; + \;k^{\prime}){\kern 1pt} \,u_{3,ii} \; + \;\lambda^{\prime}_{1} {\kern 1pt} \varphi_{,z}^{*} \; - \;k^{\prime}{\kern 1pt} \varphi_{2,x} \; - \;\beta^{\prime}_{1} {\kern 1pt} T_{,z} \; = \;\frac{{\rho \,(\;1\; - \;\eta^{2} \,\nabla^{2} \;)}}{{\alpha_{1} }}\,u_{3,tt} ,$$

11 $$\gamma^{\prime}{\kern 1pt} \varphi_{2,ii} \; + \;k^{\prime}{\kern 1pt} (u_{1,z} \; - \;u_{3,x} )\; - \;2\,k^{\prime}\,\varphi_{2} \; = \;\frac{{\rho \,J\,(\;1\; - \;\eta^{2} \,\nabla^{2} \;)}}{{\alpha_{1} }}\,\varphi_{2,tt} .$$

12 $$\alpha^{\prime}_{0} \;\varphi_{,ii}^{*} \; + \;\frac{1}{3}\;\nu^{\prime}{\kern 1pt} \,(\;T\; - T_{0} \;)\; - \;\frac{1}{3}\;\lambda^{\prime}_{1} {\kern 1pt} \varphi^{*} \; - \;\frac{1}{3}\;\lambda^{\prime}_{0} \;u_{i,i} \; = \;\frac{{3\,\rho {\kern 1pt} J_{0} \;(\;1\; - \;\eta^{2} \,\nabla^{2} \;)}}{{2\;\alpha_{1} }}\;{\kern 1pt} \varphi_{,tt}^{*} .$$

13 $$k^{*} \,T_{,ii} \; + \;(k_{1}^{*} \; + \;k^{*} \tau_{\nu } )\;T_{,t} \; + \;k_{1}^{*} \,\tau_{\theta } \,T_{,iitt} \; = \;[1\; + \;\tau_{q} \frac{\partial }{\partial t}\; + \;\frac{1}{2}\;\tau_{q}^{2} \frac{{\partial^{2} }}{{\partial t^{2} }}][\rho {\kern 1pt} c_{E} T_{,tt} \; + \;\nu^{\prime}\,\alpha_{1} {\kern 1pt} T_{0} \varphi_{,tt}^{*} \; + \;\beta^{\prime}_{1} \,\alpha_{1} T_{0} e_{,tt} \;].$$

where $$\alpha_{1} \; = \,1\; - \;\alpha^{*} \,T_{0} .$$

Inserting the following dimensionless variables

14 $$\begin{gathered} (\overline{x},\overline{z})\; = \;\frac{{\omega^{*} }}{{c_{1} }}(x,z),\,\overline{u}_{i} \; = \;\frac{{\rho \,\omega^{*} {\kern 1pt} c_{1} \,u_{i} }}{{\beta^{\prime}_{1} {\kern 1pt} T_{0} }},\,(\overline{t},\;\overline{\tau }_{\nu } ,\;\overline{\tau }_{\theta } ,\;\overline{\tau }_{q} )\; = \;\omega^{*} (t,\;\tau_{\nu } ,\;\tau_{\theta } ,\;\tau_{q} ),\,\overline{\varphi }_{2} \; = \;\frac{{\rho \,c_{1}^{2} \,\varphi_{2} }}{{\beta^{\prime}_{1} {\kern 1pt} T_{0} }}, \hfill \\ \overline{\varphi }^{*} = \;\frac{{\rho \,c_{1}^{2} {\kern 1pt} \varphi^{*} }}{{\beta^{\prime}_{1} {\kern 1pt} T_{0} }},\,\,\overline{T} = \;\frac{{T\; - \;T_{0} }}{{T_{0} }},\,\,\overline{\sigma }_{ij} \; = \;\frac{{\sigma_{ij} }}{{\beta^{\prime}_{1} {\kern 1pt} T_{0} }},\,\overline{m}_{ij} \; = \;\frac{{\omega^{*} m_{ij} }}{{c_{1} \beta^{\prime}_{1} T_{0} }},\,\overline{\lambda }_{k} \; = \;\frac{{\omega^{*} \lambda_{k} }}{{c_{1} \beta^{\prime}_{1} T_{0} }}, \hfill \\ \overline{\eta } = \;\frac{{\omega^{*} }}{{c_{1} }}\,\eta ,\,c_{1}^{2} \; = \;\frac{{\lambda^{\prime}\; + \;2\,\mu^{\prime}\; + \;k^{\prime}}}{\rho },\,\omega^{*} \; = \;\frac{{\rho \;c_{E} \;c_{1}^{2} }}{{k_{1}^{*} }}. \hfill \\ \end{gathered}$$

By adding the displacement potentials ($$\Phi ,$$$$\psi$$) that are equivalent to the displacement components, we get

15 $$u_{1} \; = \;\Phi_{,x} \; + \;\psi_{,z} ,\;\;\;\;\;u_{3} \; = \;\Phi_{,z} \; - \;\psi_{,x} .$$

From Eqs. (14) and (15) in Eqs. (9) - (13), we obtain

16 $$[\,(\;a_{1} \; + \;a_{2} )\;\nabla^{2} \; - \;\frac{{(\;1\; - \;\eta^{2} \,\nabla^{2} \;)}}{{\alpha_{1} }}\;\frac{{\partial^{2} }}{{\partial \,t^{2} }}\;]\,\Phi \; + \;a_{3} {\kern 1pt} \varphi^{*} \; - \;T\; = \;0,$$

17 $$[\,a_{2} \,\nabla^{2} \; - \;\frac{{(\;1\; - \;\eta^{2} \,\nabla^{2} \;)}}{{\alpha_{1} }}\;\frac{{\partial^{2} }}{{\partial \,t^{2} }}\,]\,\psi \; - \;a_{4} \,\varphi_{2} \; = \;0,$$

18 $$[\,\nabla^{2} \; - \;2\,a_{5} \; - \;\frac{{a_{6} \,(\;1\; - \;\eta^{2} \,\nabla^{2} \;)}}{{\alpha_{1} }}\,\frac{{\partial^{2} }}{{\partial \,t^{2} }}\,]\,\varphi_{2} \; + \;\;a_{5} \,\nabla^{2} \,\psi = \;0,$$

19 $$[\,a_{7} {\kern 1pt} \nabla^{2} \; - \;\frac{1}{3}\;a_{3} \; - \;\frac{{a_{10} \,(\;1\; - \;\eta^{2} \,\nabla^{2} \;)}}{{\alpha_{1} }}{\kern 1pt} \;\frac{{\partial^{2} }}{{\partial \,t^{2} }}\;]\,\varphi^{*} \; + \;a_{8} {\kern 1pt} T\; - \;a_{9} {\kern 1pt} \nabla^{2} \,\Phi \; = \;0,$$

20 $$\nabla^{2} \,T\; + \;(\,a_{11} \; + \;\tau_{\nu } \,)\,\nabla^{2} \,T_{,t} \; + \;a_{12} \,\nabla^{2} \,T_{,tt} \; = \;[\,1\; + \;\tau_{q} \,\frac{\partial }{\partial t}\; + \;\frac{1}{2}\;\tau_{q}^{2} \frac{{\partial^{2} }}{{\partial t^{2} }}][\;a_{13} T_{,tt} \; + \;a_{14} {\kern 1pt} \varphi_{,tt}^{*} \; + \;a_{15} \,\nabla^{2} \,\Phi_{,tt} \;].$$

## The solution

In this section, the normal mode technique used to solve the fore-mentioned five equations to determine the value of displacement potentials ($\Phi$ and $\psi$), scalar micro-stretch function ($\varphi^{*}$), micro-rotation vector ($\user2{\varphi }$), and the temperature ($T$), These quantities are then utilized to calculate the displacement components, stress tensor components, the couple stress tensor components and the micro-stress tensor components. Suppose that

21 $$[u_{i} ,\;\Phi ,\;\psi ,\;\varphi^{*} ,\;\varphi_{2} ,\;T,\;\sigma_{ij} ,\;m_{ij} ,\;u_{i}^{f} ,\;\sigma_{ij}^{f} ]\,(x,\;z,\;t)\; = \;[\overline{u}_{i} ,\;\overline{\Phi },\;\overline{\psi },\;\overline{\varphi }^{*} ,\;\overline{\varphi }_{2} ,\;\overline{T},\;\overline{\sigma }_{ij} ,\;\overline{m}_{ij} ,\;\overline{u}_{i}^{f} ,\;\overline{\sigma }_{ij}^{f} ]\;(z)\;e^{i\,b\,(\,x\, - \,\xi \,t\,)} .$$

Using Eq. (21) in Eqs. (16)-(20), we have

22 $$(\delta_{1} {\kern 1pt} \rm{D}^{2} \; + \;\delta_{2} )\;\overline{\Phi }\; + \;a_{3} {\kern 1pt} \overline{\varphi }^{*} \; - \;\overline{T}\; = \;0,$$

23 $$(\delta_{3} {\kern 1pt} \rm{D}^{2} \; + \;\delta_{4} )\;\overline{\psi }\; - \;a_{4} {\kern 1pt} \overline{\varphi }_{2} \; = \;0,$$

24 $$(a_{5} {\kern 1pt} \rm{D}^{2} \; - \;\delta_{7} )\;\overline{\psi }\; + \;(\;\delta_{5} \;\rm{D}^{2} \; + \;\delta_{6} )\;\overline{\varphi }_{2} \; = \;0,$$

25 $$(\; - a_{9} {\kern 1pt} \rm{D}^{2} \; + \;\delta_{10} {\kern 1pt} )\;\overline{\Phi }\; + \;(\;\delta_{8} {\kern 1pt} \rm{D}^{2} \; + \;\delta_{9} \,)\;\overline{\varphi }^{*} \; + \;a_{8} \,T\; = \;0,$$

26 $$(\delta_{15} {\kern 1pt} \rm{D}^{2} \; - \;\delta_{16} )\;\overline{\Phi }\; + \;\delta_{14} {\kern 1pt} \overline{\varphi }^{*} \; + \;(\delta_{12} {\kern 1pt} \rm{D}^{2} \; + \;\delta_{13} )\;\overline{T}\; = \;0,$$

For the presence of non-trivial solutions, the determinant of the above-mentioned Eqs. (22), (25), and (26) must be zero. This condition leads to the following result:

27 $$(\rm{D}^{6} \; - \;A\,\rm{D}^{4} \; + \;B\,\rm{D}^{2} \; - \;C\,)\,\{ \,\overline{\Phi }\,(z)\,,\,\overline{\varphi }^{*} \,(z)\,,\;\overline{T}\,(z)\,\} \; = \;0.$$

Equation (27) becomes

28 $$(\rm{D}^{2} - k_{1}^{2} )\,(\rm{D}^{2} - k_{2}^{2} )\,(\rm{D}^{2} - k_{3}^{2} )\;\{ \overline{\Phi }\,(z)\,,\,\overline{\varphi }^{*} \,(z),\;\overline{T}\,(z)\} = 0.$$

where, $k_{n}^{2} ,\,(\;n\; = \;1,\;2,\;3\;)$ are the roots of the auxiliary equation of Eq. (28). Then

29 $$\overline{\Phi }{\kern 1pt} {\kern 1pt} (z)\; = \;\sum\limits_{n\, = \,1}^{3} {M_{n} \;e^{{k_{n} \,z}} } \; + \;\sum\limits_{n\, = \,1}^{3} {M_{n + 3} \;e^{{ - k_{n} \,z}} } ,$$

30 $$\overline{\varphi }^{*} \,(z)\; = \;\sum\limits_{n\, = \,1}^{3} {H_{1n} M_{n} \;e^{{k_{n} \,z}} } \; + \;\sum\limits_{n\, = \,1}^{3} {H_{1\,(\,n\, + \,3\,)} M_{n + 3} \;e^{{ - k_{n} \,z}} } ,$$

31 $$\overline{T}\,(z)\; = \;\sum\limits_{n\, = \,1}^{3} {H_{2n} M_{n} \;e^{{k_{n} \,z}} } \; + \;\sum\limits_{n\, = \,1}^{3} {H_{2\,(\,n\, + \,3\,)} M_{n + 3} \;e^{{ - k_{n} \,z}} } .$$

For the presence of non-trivial solutions, the determinant of the above-mentioned Eqs. (23) and (24) must be zero. This condition leads to the following result:

32 $$(\rm{D}^{4} \; - \;E\,\rm{D}^{2} \; + \;F\,)\,\{ \,\overline{\psi }\;(z)\,,\,\overline{\varphi }_{2} \,(z)\,\} \; = \;0.$$

Equation (32) reduces to

33 $$(D^{2}-S_{l}^{2})(D^{2}-S_{2}^{2})\,\{ \,\overline{\psi }\;(z)\,,\,\overline{\varphi }_{2} \,(z)\,\} \; = \;0.$$

where, $S_{l}^{2} ,\,(\;l\; = \;1,\;2\;)$ are the roots of the auxiliary equation of Eq. (33). Then

34 $$\overline{\psi }\;(z)\; = \;\sum\limits_{l = \,1}^{2} {N_{l} \;e^{{S_{l} \,z}} } \; + \;\sum\limits_{l\, = \,1}^{2} {N_{l\, + \,2} \;e^{{ - S_{l} \,z}} } ,$$

35 $$\overline{\varphi }_{2} (z)\; = \;\sum\limits_{l\, = \,1}^{2} {H_{3l} \;N_{l} \;e^{{S_{l} \,z}} } \; + \;\sum\limits_{l\, = \,1}^{2} {H_{3\,(\,l\, + \,2\,)} \;N_{l + 2} \;e^{{ - S_{l} \,z}} } ,$$

Using Eq. (21) into Eq. (15), then utilizing Eqs. (29) and (34) we acquire

36 $$\overline{u}_{1} (z)\; = \;\sum\limits_{n\, = \,1}^{3} {\,i\;b\,M_{n} \,e^{{k_{n} \,z}} } \; + \;\sum\limits_{n\, = \,1}^{3} {\,i\;{\kern 1pt} b\;M_{n + 3} \;e^{{ - \,k_{n} \,z}} } \; + \;\sum\limits_{l\, = \,1}^{2} {\,S_{l} \;N_{l} \;e^{{S_{l} \,z}} } \, - \;\sum\limits_{l\, = \,1}^{2} {\,S_{l} \;N_{l\, + \,2} \;e^{{ - \,S_{l} \,z}} } ,$$

37 $$\overline{u}_{3} (z)\; = \;\sum\limits_{n\, = \,1}^{3} {\,k_{n} \;M_{n} \,e^{{k_{n} \,z}} } \; - \;\sum\limits_{n\, = \,1}^{3} {\,k_{n} \;M_{n + 3} \;e^{{ - \,k_{n} \,z}} } \; - \;\sum\limits_{l\, = \,1}^{2} {\,i\;b\;N_{l} \;e^{{S_{l} \,z}} } \, - \;\sum\limits_{l\, = \,1}^{2} {\,i\;b\;N_{l\, + \,2} \;e^{{ - \,S_{l} \,z}} } .$$

By compensation from Eqs. (14) and (21) in Eqs. (1) and by using Eqs. (30), (31), (35), (36) and (37), we realize that

38 $$\overline{\sigma }_{xx} (z)\; = \;\sum\limits_{n\, = \,1}^{3} {H_{4n} \;M_{n} \;e^{{k_{n} \,z}} } \; + \;\sum\limits_{n\, = \,1}^{3} {H_{4\,(\,n\, + \,3\,)} \;M_{n + 3} \;e^{{ - \,k_{n} \,z}} } \, + \,\sum\limits_{l\, = \,1}^{2} {H_{5l} \;N_{l} \;e^{{S_{l} \,z}} } \; + \;\sum\limits_{l\, = \,1}^{2} {H_{5\,(\,l\, + \,2\,)} \;N_{l + 2} \;e^{{ - \,S_{l} \,z}} } ,$$

39 $$\overline{\sigma }_{yy} (z)\; = \;\sum\limits_{n\, = \,1}^{3} {H_{6n} M_{n} \;e^{{k_{n} \,z}} } \; + \;\sum\limits_{n\, = \,1}^{3} {H_{6\,(\,n\, + \,3\,)} M_{n + 3} \;e^{{ - k_{n} \,z}} } ,$$

40 $$\overline{\sigma }_{zz} (z)\; = \;\sum\limits_{n\, = \,1}^{3} {H_{7n} \;M_{n} \;e^{{k_{n} \,z}} } \; + \;\sum\limits_{n\, = \,1}^{3} {H_{7\,(\,n\, + \,3\,)} \;M_{n + 3} \;e^{{ - \,k_{n} \,z}} } \, + \,\sum\limits_{l\, = \,1}^{2} {H_{8l} \;N_{l} \;e^{{S_{l} \,z}} } \; + \;\sum\limits_{{n^{\prime}\, = \,1}}^{2} {H_{8\,(\,l\, + \,2\,)} \;N_{l + 2} \;e^{{ - \,S_{{n^{\prime}}} \,z}} } ,$$

41 $$\overline{\sigma }_{xz} (z)\; = \;\sum\limits_{n\, = \,1}^{3} {H_{9n} \;M_{n} \;e^{{k_{n} \,z}} } \; + \;\sum\limits_{n\, = \,1}^{3} {H_{9\,(\,n\, + \,3\,)} \;M_{n + 3} \;e^{{ - \,k_{n} \,z}} } \, + \,\sum\limits_{l\, = \,1}^{2} {H_{10l} \;N_{l} \;e^{{S_{l} \,z}} } \; + \;\sum\limits_{l\, = \,1}^{2} {H_{10\,(\,l\, + \,2\,)} \;N_{l + 2} \;e^{{ - \,S_{l} \,z}} } ,$$

42 $$\overline{\sigma }_{zx} (z)\; = \;\sum\limits_{n\, = \,1}^{3} {H_{9n} \;M_{n} \;e^{{k_{n} \,z}} } \; + \;\sum\limits_{n\, = \,1}^{3} {H_{9(\,n\, + \,3\,)} \;M_{n + 3} \;e^{{ - \,k_{n} \,z}} } \, + \,\sum\limits_{l\, = \,1}^{2} {H_{11l} \;N_{l} \;e^{{S_{l} \,z}} } \; + \;\sum\limits_{l\, = \,1}^{2} {H_{11\,(\,l\, + \,2\,)} \;N_{l + 2} \;e^{{ - \,S_{l} \,z}} } .$$

By compensation from Eqs. (14) and (21) into Eqs. (2) and (3) and using Eqs. (30) and (35) the couple stress tensor components and the micro-stress tensor have the form

43 $$\overline{m}_{xy} (z)\; = \;\sum\limits_{l\, = \,1}^{2} {H_{12l} \,N_{l} \;e^{{S_{l} \,z}} } \; + \;\sum\limits_{l\, = \,1}^{2} {H_{12\,(\,l\, + \,2\,)} \,N_{l + 2} \;e^{{ - S_{l} \,z}} } ,$$

44 $$\overline{m}_{yx} (z)\; = \;\sum\limits_{l\, = \,1}^{2} {H_{14l} \,N_{l} \;e^{{S_{l} \,z}} } \; + \;\sum\limits_{l\, = \,1}^{2} {H_{14\,(\,l\, + \,2\,)} \,N_{l + 2} \;e^{{ - S_{l} \,z}} } ,$$

45 $$\overline{m}_{zy} (z)\; = \;\sum\limits_{l\, = \,1}^{2} {H_{13l} \,N_{l} \;e^{{S_{l} \,z}} } \; + \;\sum\limits_{l\, = \,1}^{2} {H_{13\,(\,l\, + \,2\,)} \,N_{l + 2} \;e^{{ - S_{l} \,z}} } ,$$

46 $$\overline{m}_{yz} (z)\; = \;\sum\limits_{l\, = \,1}^{2} {H_{15l} \,N_{l} \;e^{{S_{l} \,z}} } \; + \;\sum\limits_{l\, = \,1}^{2} {H_{15\,(\,l\, + \,2\,)} \,N_{l + 2} \;e^{{ - S_{l} \,z}} } ,$$

47 $$\overline{\lambda }_{x} (z)\; = \;\sum\limits_{n\, = \,1}^{3} {H_{16n} \;M_{n} \;e^{{k_{n} \,z}} } \; + \;\sum\limits_{n\, = \,1}^{3} {H_{16\,(\,n\, + \,3\,)} \;M_{n + 3} \;e^{{ - k_{n} \,z}} } ,$$

48 $$\overline{\lambda }_{z} (z)\; = \;\sum\limits_{n\, = \,1}^{3} {H_{17n} \;M_{n} \;e^{{k_{n} \,z}} } \; + \;\sum\limits_{n\, = \,1}^{3} {H_{17\,(\,n\, + \,3\,)} M_{n + 3} \;e^{{ - k_{n} \,z}} } .$$

Within the fluid scenario, the governing equations are formulated as follows [30. 31]

49 $$\lambda^{f} \,\nabla \,(\,\nabla \,.\,{\varvec{u}}^{f} \,)\, = \,\rho^{f} \,{\varvec{u}}_{,tt}^{f} ,$$

50 $$\sigma_{ij}^{f} \, = \,\lambda^{f} \,u_{r,r}^{f} \,\delta_{ij} .$$

Substituting from Eq. (21) in Eqs. (49) and (50)

51 $$(\,\frac{{\omega^{2} {\kern 1pt} b^{2} }}{{c_{1}^{{f^{2} }} }}\; - \;b^{2} )\,\overline{u}_{1}^{f} \, + \,i\,b\,\rm{D }\overline{u}_{3}^{f} \, = \,0,$$

52 $$(\rm{D}^{2} \, + \,\frac{{\omega^{2} {\kern 1pt} b^{2} }}{{c_{1}^{{f^{2} }} }})\,\overline{u}_{3}^{f} \, + \,i\,b\,\rm{D }\overline{u}_{1}^{f} \, = \,0.$$

where, $$c_{1}^{{f^{2} }} \, = \,\frac{{\lambda^{f} }}{{\rho^{f} }}.$$

Eliminating $\overline{u}_{1}^{f} ,\,\;\overline{u}_{3}^{f}$ between Eqs. (51) and (52), we obtain

53 $$[\;\rm{D}^{2} \; - \;r^{2} \;]\;(\,\overline{u}_{1}^{f} ,\;\overline{u}_{3}^{f} \,)\, = \,0.$$

where $r^{2} \; = \;(\;b^{2} \; - \;\frac{{\omega^{2} \;b^{2} }}{{c_{1}^{{f^{2} }} }}\;),$ is the root of the auxiliary equation of Eq. (53), the solution of Eq. (53) becomes

54 $$(\,\overline{u}_{1}^{f} ,\;\overline{u}_{3}^{f} )\,(z)\, = \,(1,\;L_{11} )\,R_{1} \,e^{r\,z} \, + \,(1,\;L_{12} )\,R_{2} \,e^{ - \,r\,z} .$$

Utilizing Eq. (21) in Eq. (50) and with aid Eq. (54), one receives that

55 $$\overline{\sigma }_{xx}^{f} (z)\, = \,\overline{\sigma }_{yy}^{f} (z)\, = \,\overline{\sigma }_{zz}^{f} (z)\, = \,L_{21} R_{1} \,e^{r\,z} \, + \,L_{2\,2} R_{2} \,e^{ - \,r\,z} .$$

$$L_{1\,1} \, = \,\frac{{i{\kern 1pt} b{\kern 1pt} r}}{{[r^{2} \; + \;\frac{{\omega^{2} {\kern 1pt} b^{2} }}{{c_{1}^{{f^{2} }} }}]}},\,L_{1\,2} \, = \,\frac{{ - \;i{\kern 1pt} {\kern 1pt} b{\kern 1pt} r}}{{[r^{2} \; + \;\frac{{\omega^{2} {\kern 1pt} b^{2} }}{{c_{1}^{{f^{2} }} }}]}},\,L_{2\,1} \; = \;\lambda^{f} [i{\kern 1pt} b\; + \;r{\kern 1pt} L_{1\,1} ],\,L_{2\,2} \; = \;\lambda^{f} [i{\kern 1pt} b\; - \;r{\kern 1pt} L_{1\,2} ].$$

## The boundary conditions

To find the constants $M_{1} ,\,M_{2} ,M_{3} ,M_{4} ,M_{5} ,M_{6} ,\,N_{1} ,\,N_{2} ,N_{3} ,N_{4} ,\,\,R_{1}$ and $R_{2} ,$ we have applied the boundary conditions for the problem at $z\; = \; \pm \;d$,

56 $$\sigma_{xx} \; = \;\sigma_{xx}^{f} , \,\sigma_{xz} \; = \;f_{1} \;e^{i\;b\;(\;x\; - \;\xi \;t\;)} ,\,\frac{{\partial \;u_{1} }}{\partial \;z}\; = \;\frac{{\partial \;u_{1}^{f} }}{\partial \;z},\,\varphi^{ * } \; = \;0,\,T\; = \;f_{2} \;e^{i\;b\;(\;x\; - \;\xi \;t\;)} ,\,\varphi_{2} \; = \;0,\,{\text{at}}\,z\; = \; \pm \;d$$

Using the expressions for $\sigma_{xx} ,\;{\kern 1pt} \sigma_{xx}^{f} ,\;\sigma_{xz} ,\;{\kern 1pt} T,\;{\kern 1pt} u_{1} ,\;{\kern 1pt} u_{1}^{f} ,\;{\kern 1pt} \varphi^{ * }$ and $\varphi_{2}$ in (56), we get

57 $$\sum\limits_{n\, = \,1}^{3} {H_{4\,n} \,M_{n} e^{{k_{n} \,d}} } + \sum\limits_{n\, = \,1}^{3} {H_{4\,(\,n\, + \,3\,)} M_{n + 3} \;e^{{ - \,k_{n} \,d}} } \, + \,\sum\limits_{l\, = \,1}^{2} {H_{5l} N_{l} \;e^{{S_{l} \,d}} } + \;\sum\limits_{l\, = \,1}^{2} {H_{5\,(\,l\, + \,2\,)} N_{l + 2} \;e^{{ - \,S_{l} \,d\,}} } - \;L_{2\,2} R_{2} \;e^{ - \,r\,d} \; = \;0,$$

58 $$\sum\limits_{n\, = \,1}^{3} {H_{4\,n} \,M_{n} e^{{ - k_{n} \,d}} } + \sum\limits_{n\, = \,1}^{3} {H_{4\,(\,n\, + \,3\,)} M_{n + 3} \;e^{{k_{n} \,d}} } \, + \,\sum\limits_{l\, = \,1}^{2} {H_{5l} N_{l} \;e^{{ - S_{l} \,d}} } + \;\sum\limits_{l\, = \,1}^{2} {H_{5\,(\,l\, + \,2\,)} N_{l + 2} \;e^{{S_{l} \,d\,}} } - \;L_{2\,1} R_{1} \;e^{ - \,r\,d} \; = \;0,$$

59 $$\sum\limits_{n\, = \,1}^{3} {H_{9\,n} \,M_{n} e^{{k_{n} \,d}} } + \sum\limits_{n\, = \,1}^{3} {H_{9\,(\,n\, + \,3\,)} M_{n + 3} \;e^{{ - \,k_{n} \,d}} } \, + \,\sum\limits_{l\, = \,1}^{2} {H_{10l} N_{l} \;e^{{S_{l} \,d}} } + \;\sum\limits_{l\, = \,1}^{2} {H_{10\,(\,l\, + \,2\,)} N_{l + 2} \;e^{{ - \,S_{l} \,d\,}} } \; = \;f_{1} ,$$

60 $$\sum\limits_{n\, = \,1}^{3} {H_{9\,n} \,M_{n} e^{{ - k_{n} \,d}} } + \sum\limits_{n\, = \,1}^{3} {H_{9\,(\,n\, + \,3\,)} M_{n + 3} \;e^{{k_{n} \,d}} } \, + \,\sum\limits_{l\, = \,1}^{2} {H_{10l} N_{l} \;e^{{ - S_{l} \,d}} } + \;\sum\limits_{l\, = \,1}^{2} {H_{10\,(\,l\, + \,2\,)} N_{l + 2} \;e^{{S_{l} \,d\,}} } \; = \;f_{1} ,$$

61 $$\sum\limits_{n\, = \,1}^{3} {i\,b\,k_{n} M_{n} \,e^{{k_{n} \,d}} } - \sum\limits_{n\, = \,1}^{3} {i\,b\,k_{n} M_{n + 3} \,e^{{ - \,k_{n} \,d}} } \, + \,\sum\limits_{l\, = \,1}^{2} {S_{l}^{2} \,N_{l} \,e^{{S_{l} \,d}} } + \;\sum\limits_{l\, = \,1}^{2} {H_{10\,(\,l\, + \,2\,)} N_{l + 2} \;e^{{ - \,S_{l} \,d\,}} } \, + \,r\,R_{2} \,e^{ - r\,d} \; = \,0,$$

62 $$\sum\limits_{n\, = \,1}^{3} {i\,b\,k_{n} M_{n} \,e^{{ - k_{n} \,d}} } - \sum\limits_{n\, = \,1}^{3} {i\,b\,k_{n} M_{n + 3} \,e^{{\,k_{n} \,d}} } \, + \,\sum\limits_{l\, = \,1}^{2} {S_{l}^{2} \,N_{l} \,e^{{ - S_{l} \,d}} } + \;\sum\limits_{l\, = \,1}^{2} {H_{10\,(\,l\, + \,2\,)} N_{l + 2} \;e^{{\,S_{l} \,d\,}} } \, - \,r\,R_{1} \,e^{ - r\,d} \; = \,0,$$

63 $$\sum\limits_{n\, = \,1}^{3} {H_{1n} M_{n} \;e^{{k_{n} \,d}} } \; + \;\sum\limits_{n\, = \,1}^{3} {H_{1\,(\,n\, + \,3\,)} M_{n + 3} \;e^{{ - k_{n} \,d}} } \; = \;0,$$

64 $$\sum\limits_{n\, = \,1}^{3} {H_{1n} M_{n} \;e^{{ - k_{n} \,d}} } \; + \;\sum\limits_{n\, = \,1}^{3} {H_{1\,(\,n\, + \,3\,)} M_{n + 3} \;e^{{k_{n} \,d}} } \; = \;0,$$

65 $$\sum\limits_{n\, = \,1}^{3} {H_{2n} M_{n} \;e^{{k_{n} \,d}} } \; + \;\sum\limits_{n\, = \,1}^{3} {H_{2\,(\,n\, + \,3\,)} M_{n + 3} \;e^{{ - \,k_{n} \,d}} } \; = \;f_{2} ,$$

66 $$\sum\limits_{n\, = \,1}^{3} {H_{2n} M_{n} \;e^{{ - k_{n} \,d}} } \; + \;\sum\limits_{n\, = \,1}^{3} {H_{2\,(\,n\, + \,3\,)} M_{n + 3} \;e^{{\,k_{n} \,d}} } \; = \;f_{2} ,$$

67 $$\sum\limits_{l\, = \,1}^{2} {H_{3l} M_{l} {\kern 1pt} e^{{S_{l} \,d}} } \; + \;\sum\limits_{l\, = \,1}^{2} {H_{3\,(\,l\, + \,2\,)} M_{l + 2} \;e^{{ - S_{l} \,d}} } \; = \;0,$$

68 $$\sum\limits_{l\, = \,1}^{2} {H_{3l} M_{l} {\kern 1pt} e^{{ - S_{l} \,d}} } \; + \;\sum\limits_{l\, = \,1}^{2} {H_{3\,(\,l\, + \,2\,)} M_{l + 2} \;e^{{S_{l} \,d}} } \; = \;0,$$

The values of constants $M_{1} ,\,M_{2} ,M_{3} ,M_{4} ,M_{5} ,M_{6} ,\,N_{1} ,\,N_{2} ,N_{3} ,N_{4} ,\,\,R_{1}$ and $R_{2} ,$ can be achieved by solving the above system of non-homogeneous equations.

## Numerical results and discussions

The analysis is performed on magnesium crystal-like material^30  ^

$$\rho \; = \;1.47\; \times \;10^{3} \;\rm{kg}\,.\,\rm{m}^{ - \,3} ,$$ $$\lambda^{\prime}\; = \;9.4\; \times \;10^{10} \;\rm{N}\,.\,\rm{m}^{ - \,2} ,$$ $$\mu^{\prime}\; = \;4\; \times \;10^{10} \;\rm{N}\,.\,\rm{m}^{ - \,2} ,$$ $$j\; = \;0.2\; \times \;10^{ - \,19} \;\rm{m}^{\,2} ,$$

$$j_{0} \; = \;1.85\; \times \;10^{ - \,19} \;\rm{m}^{\,2} ,$$ $$k^{\prime}\; = \;1\; \times \;10^{10} \;\rm{N}\,.\,\rm{m}^{ - \,2} ,$$ $$\alpha^{\prime}_{0} \; = \;0.779\; \times \;10^{ - \,9} \;\rm{N},$$ $$\gamma^{\prime}\; = \;0.779\; \times \;10^{ - \,9} \;\rm{N},$$ 

$$T_{0} \; = \;298^{ \circ } \;\rm{K},$$ $$\lambda^{\prime}_{0} \; = \;0.5\; \times \;10^{10} \;\rm{N}\,.\,\rm{m}^{ - \,2} ,$$ $$\beta^{\prime}_{1} \; = \;2.68\; \times \;10^{6} \;\rm{N}\,.\,\rm{m}^{ - \,2} \,.\,\rm{k}^{ - 1} ,$$ $$\nu^{\prime}\; = \;2\; \times \;10^{6} \;\rm{N}\,.\,\rm{m}^{ - \,2} \,.\,\rm{k}^{ - 1} ,$$ 

$$c_{E} \; = \;1.04\; \times \;10^{3} \;\rm{J}\,.\,\rm{kg}^{ - \,1} \,.\,\rm{k}^{ - 1} ,$$ $$k_{1}^{ * } \; = \;1.7\; \times \;10^{2} \;\rm{J}\,.\,\rm{m}^{ - \,1} \,.\;\rm{s}^{ - 1} .\,\rm{k}^{ - 1} ,$$ $$\tau_{\nu } \; = \;0.0171\,\rm{s,}$$ $$\tau_{\theta } \; = \;0.031\,\rm{s,}$$ 

$$\tau_{q} \; = \;0.5\,\rm{s,}$$ $$\omega_{0} \; = \;2.9,$$ $$\alpha^{*} \; = \;5 \times 10^{ - 4} \rm{K}^{ - 1} ,$$ $$\omega \; = \;\omega_{0} \; + \;i\;\zeta ,$$ 

 $$b\; = \;1,$$ $$d\; = \;1\rm{,}$$ $$f_{1} \; = \;0.0201,$$ $$f_{2} \; = \;1.0502.$$ 

Othman et al.^23^ provided the physical constants for water as a non-viscous fluid.

$$\lambda^{f} \; = \;2.25\; \times \;10^{9} \;\rm{N}\,.\,\rm{m}^{ - 2} ,\,\rho^{f} \; = \;10^{3} \;\rm{kg}\,.\,\rm{m}^{ - \,3} .$$

This study involves performing calculations for the non-dimensional value $t\, = \;0.11$ within the specified range $- 1\; \le \;z\; \le \;1$ on the surface $x = 2.18$ of each physical quantity. The presented numerical method serves to elucidate how the physical quantities $u_{1}$, $u_{3}$, $\varphi^{ * }$, $\varphi_{2}$ and $\sigma_{xz}$ change with $z.$ The graphs illustrate the principles of (G-N III) theory and.

the predicted curves of the (3PHL) model.

**Fig. 2:**
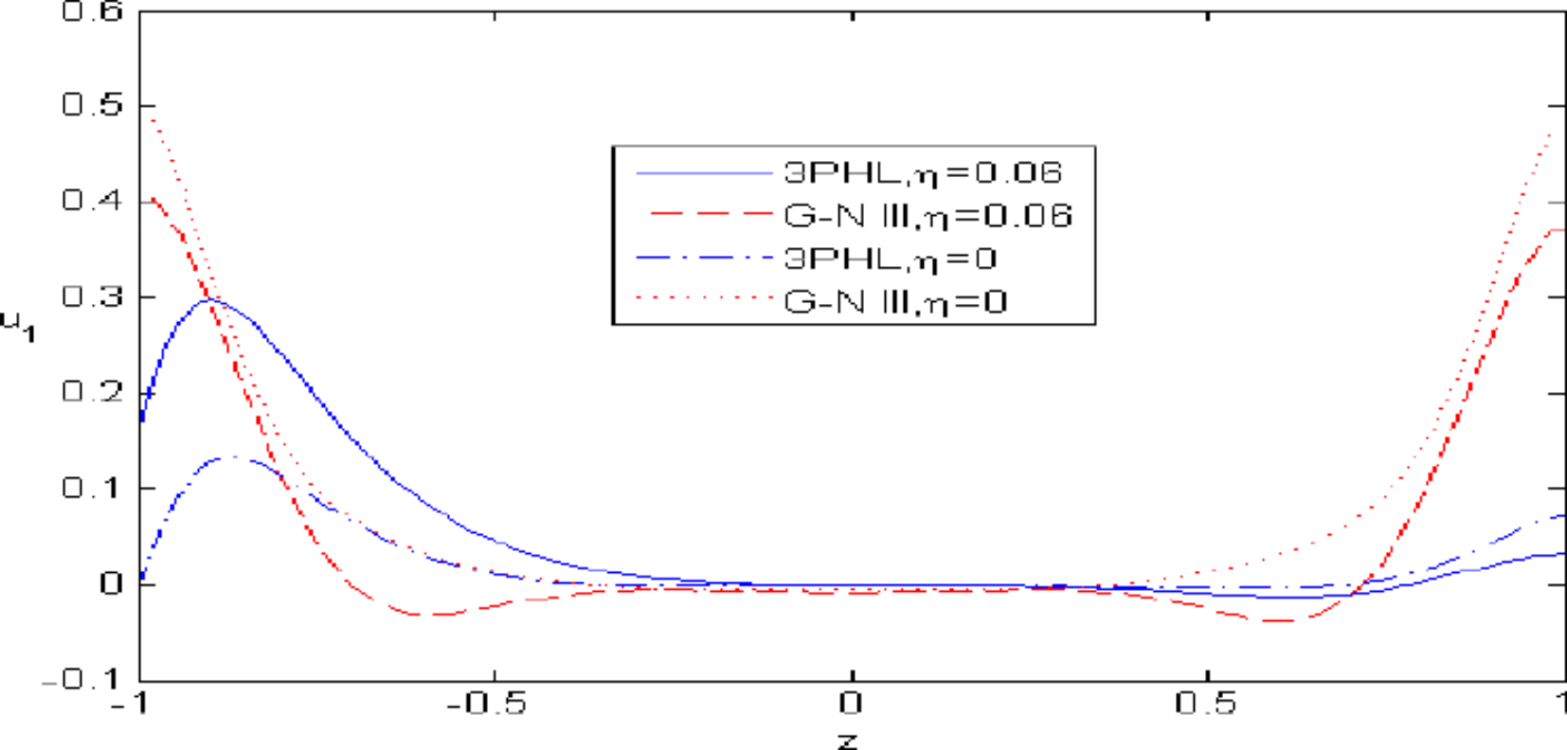
Distribution of $u_{1}$ with $z.$.

**Fig. 3:**
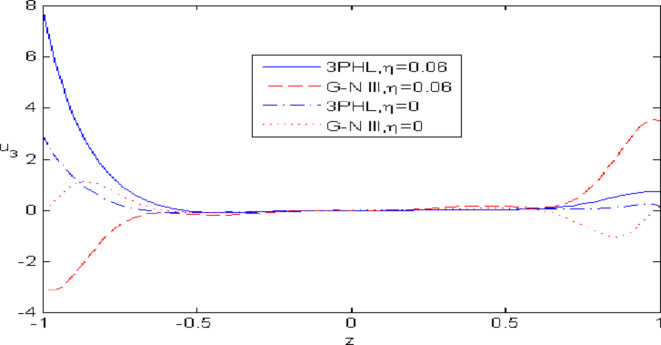
Distribution of $u_{3}$ with $z.$.

**Fig. 4:**
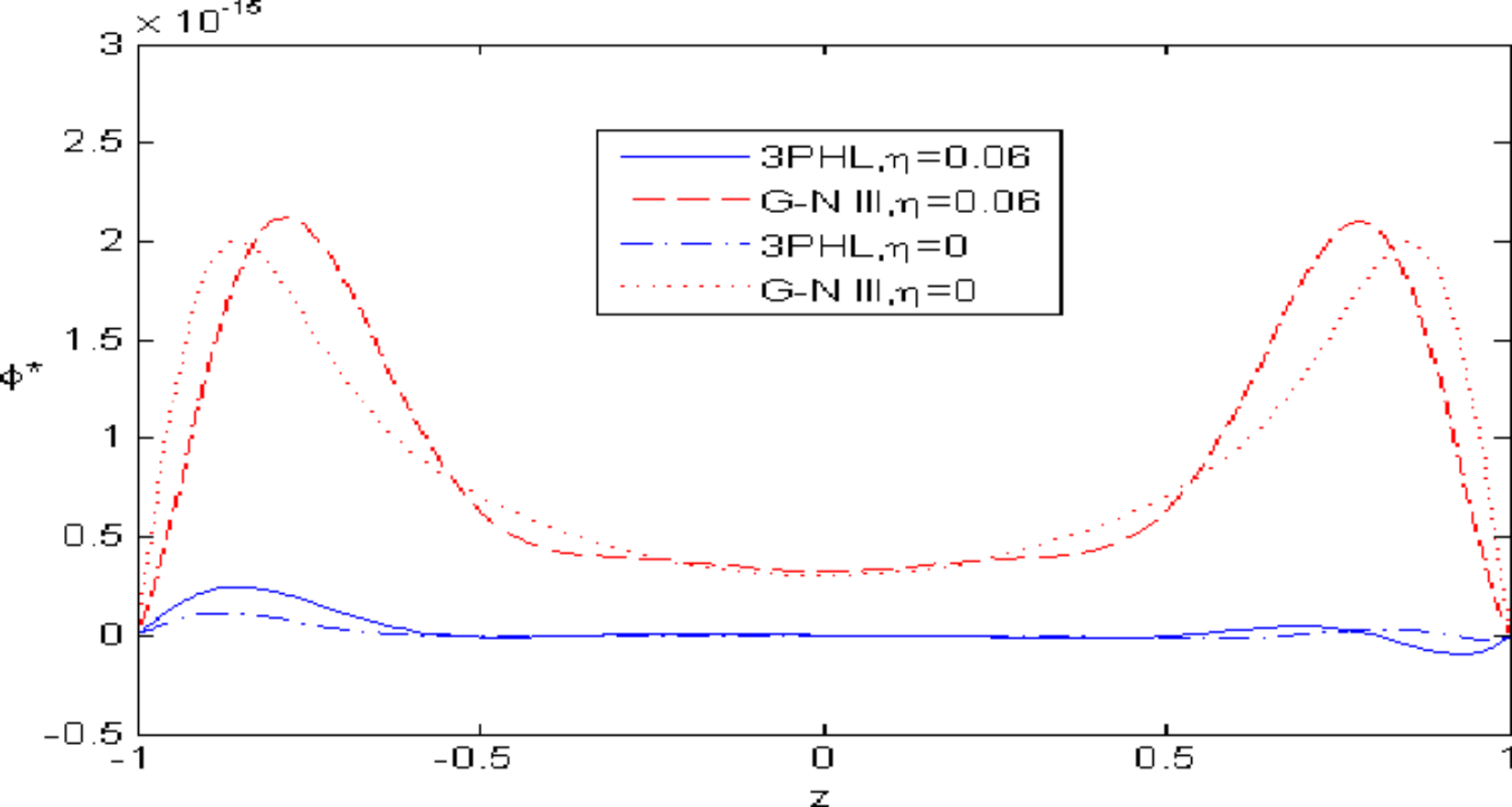
Distribution of $\varphi *$ with $z.$.

**Fig. 5:**
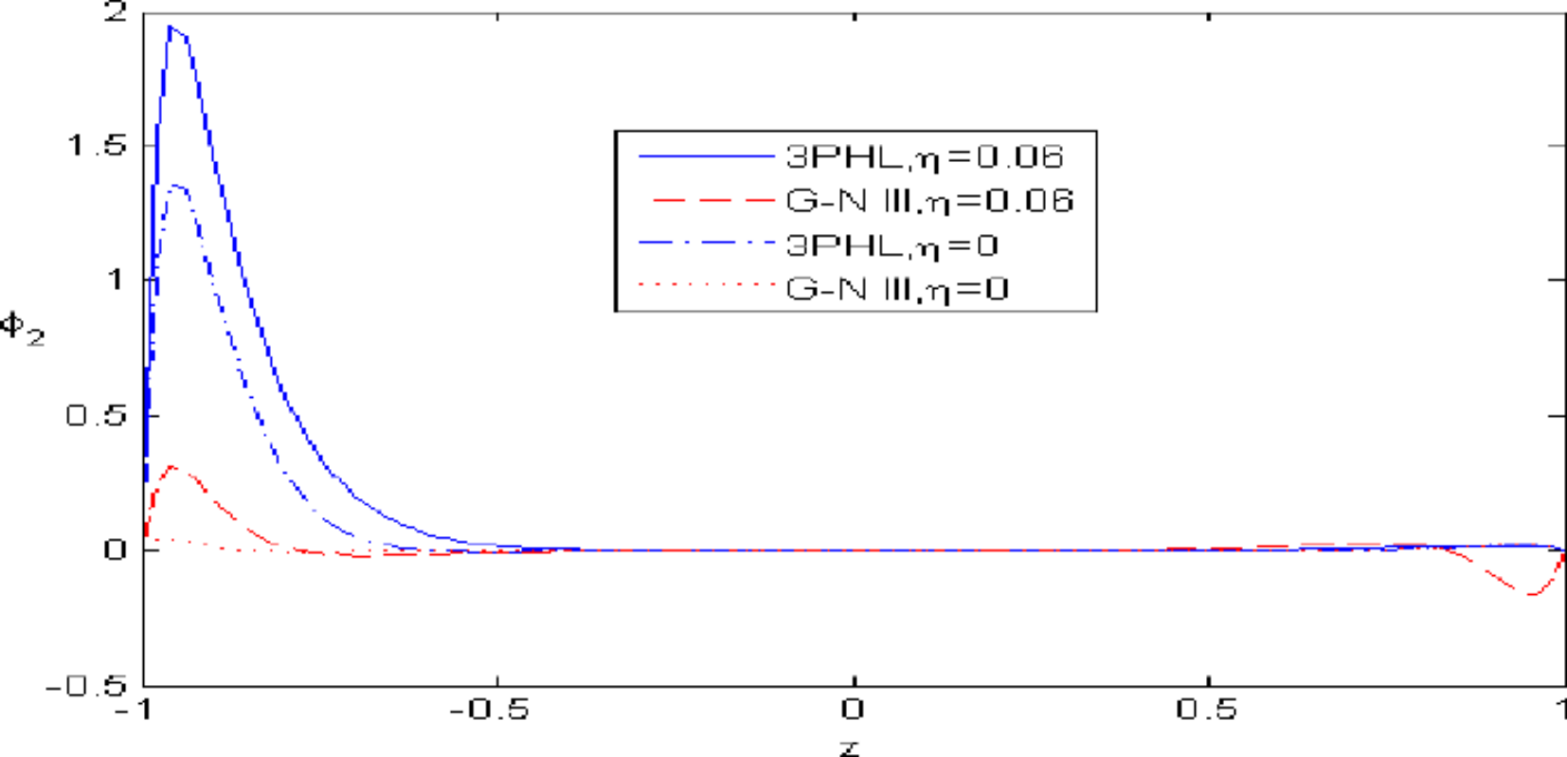
Distribution of $\varphi_{2}$ with $z.$.

**Fig. 6:**
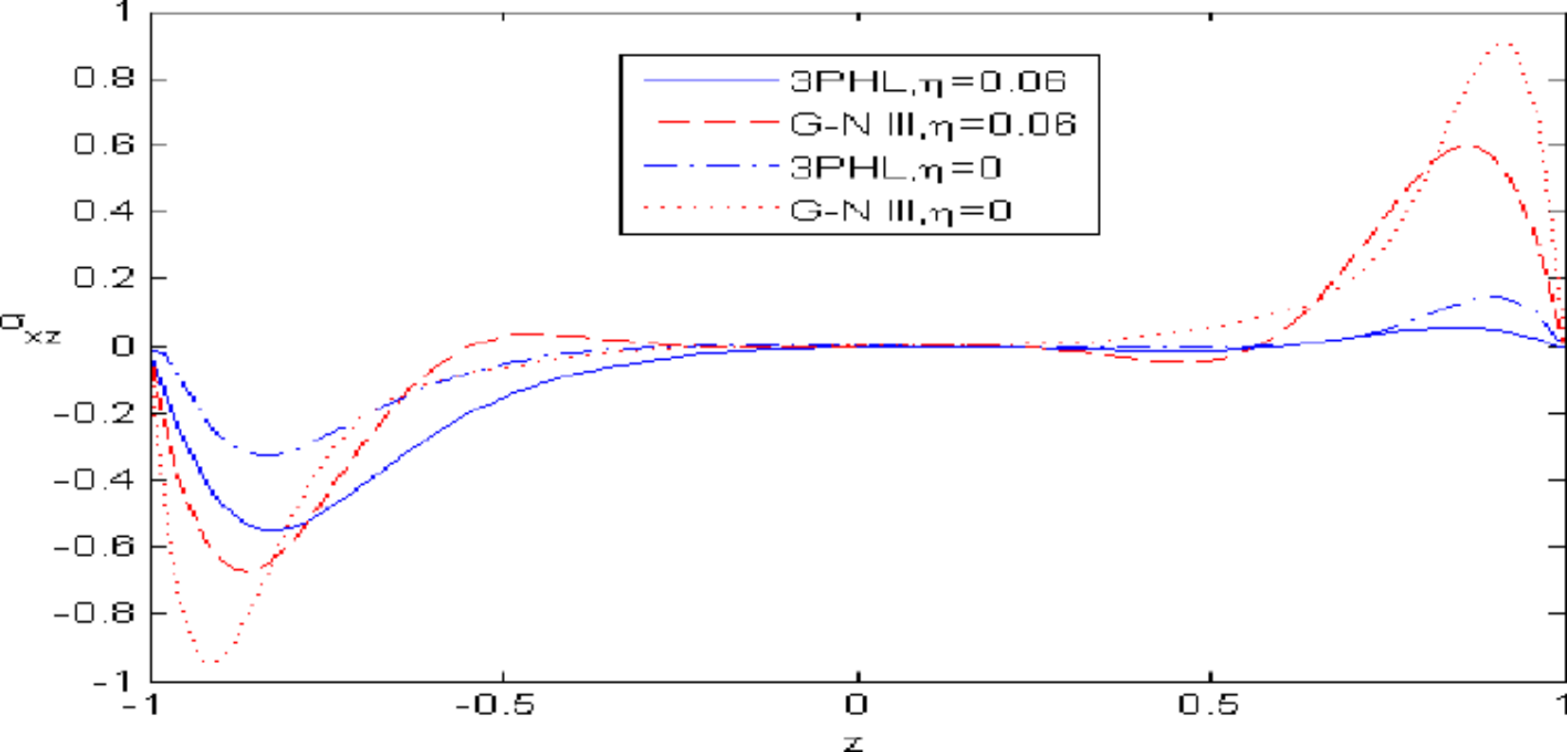
Distribution of $\sigma_{xz}$ with $z.$.

Figures 2,3,4,5,6 present a among between the (G-N III) and the (3PHL) model in scenarios with and without nonlocal effects. Figure 2 depicts the variation of $u_{1}$ with respect to $z.$ When nonlocal effects are present, the values of $u_{1}$ are lower compared to the values obtained in the absence of nonlocal effects in both theories. Figure 3 illustrates the change of $u_{3}$ against $z.$ The values of $u_{3}$ based on the (G-N III) start at positive values then decrease up to vanish at $z = 0$ then increase, while the opposite occurs in 3PHL model. Figure 4 shows the influence of nonlocal on the change of scalar micro-stretch $\varphi^{ * }$ with $z.$ It is evident that the values of $\varphi^{ * } ,$ as predicted by the (3PHL) model, are higher than the corresponding values obtained from the (G-N III) theory in both cases $(\eta \;\rm{ = }\;\rm{0,}\;0.06)$ along $z.$ Figure 5 describes the impact of nonlocal on the distribution of the micro-rotation $\varphi_{2}$ against $z.$ The four curves start from zero and increase to a maximum value, then decrease up to zero along $z$ except the value of $\varphi_{2}$ based on (3PHL) model in the presence of nonlocal. Figure 6 clarifies the change of $\sigma_{xz}$ against $z.$ It is clarified that in the presence of nonlocal, the values of $\sigma_{xz}$ based on (G-N III) are lower than its values in the absence of nonlocal along $z.$

**Fig. 7:**
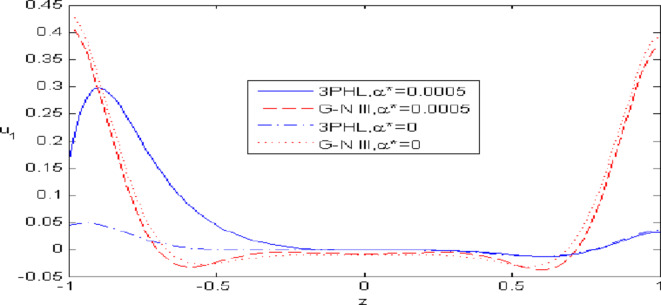
Comparing between dependent and independent temperature on $u_{1} .$.

**Fig. 8:**
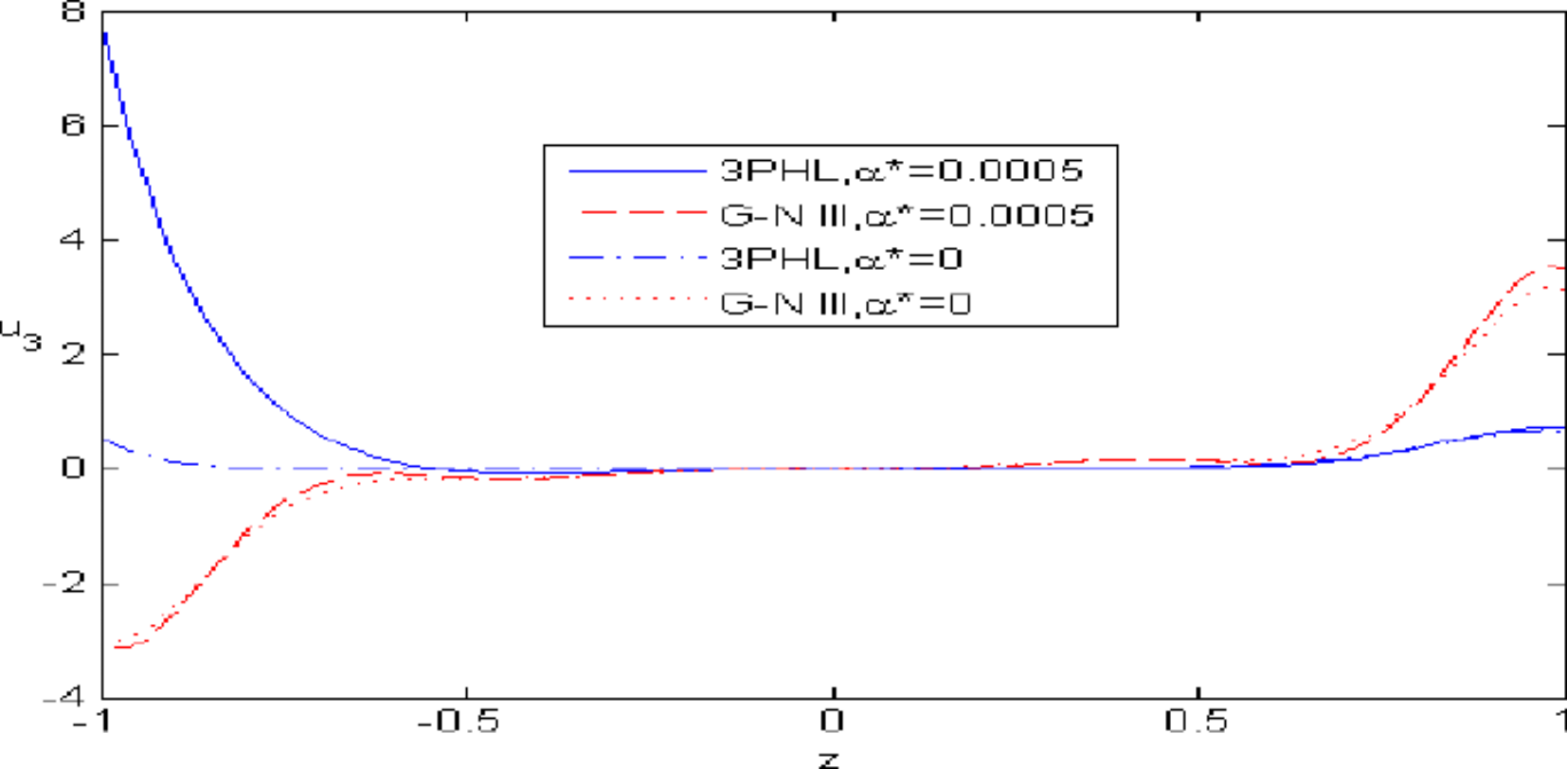
Comparing between dependent and independent temperature on $u_{3} .$.

**Fig. 9:**
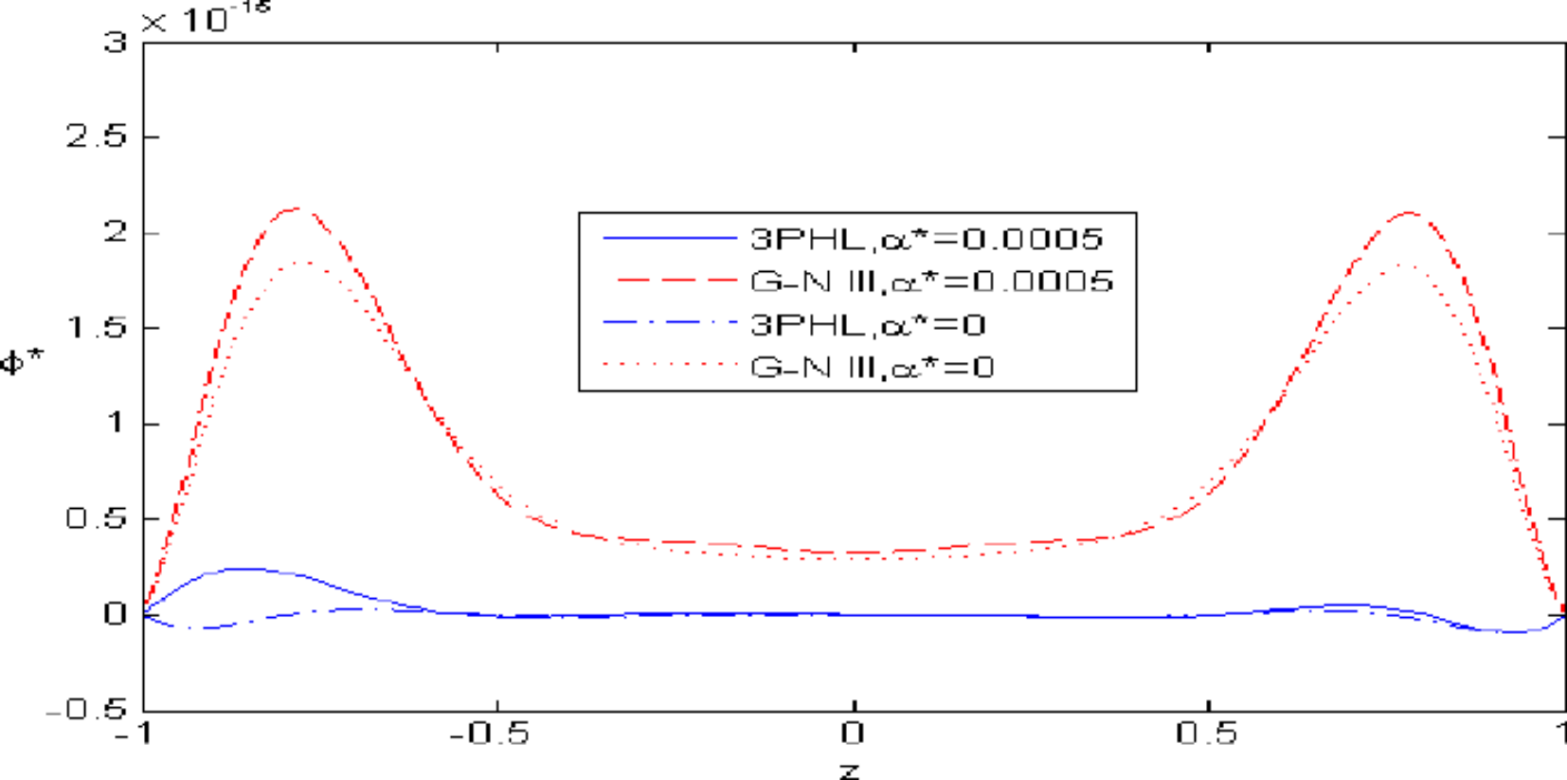
Comparing between dependent and independent temperature on $\varphi * .$.

**Fig. 10:**
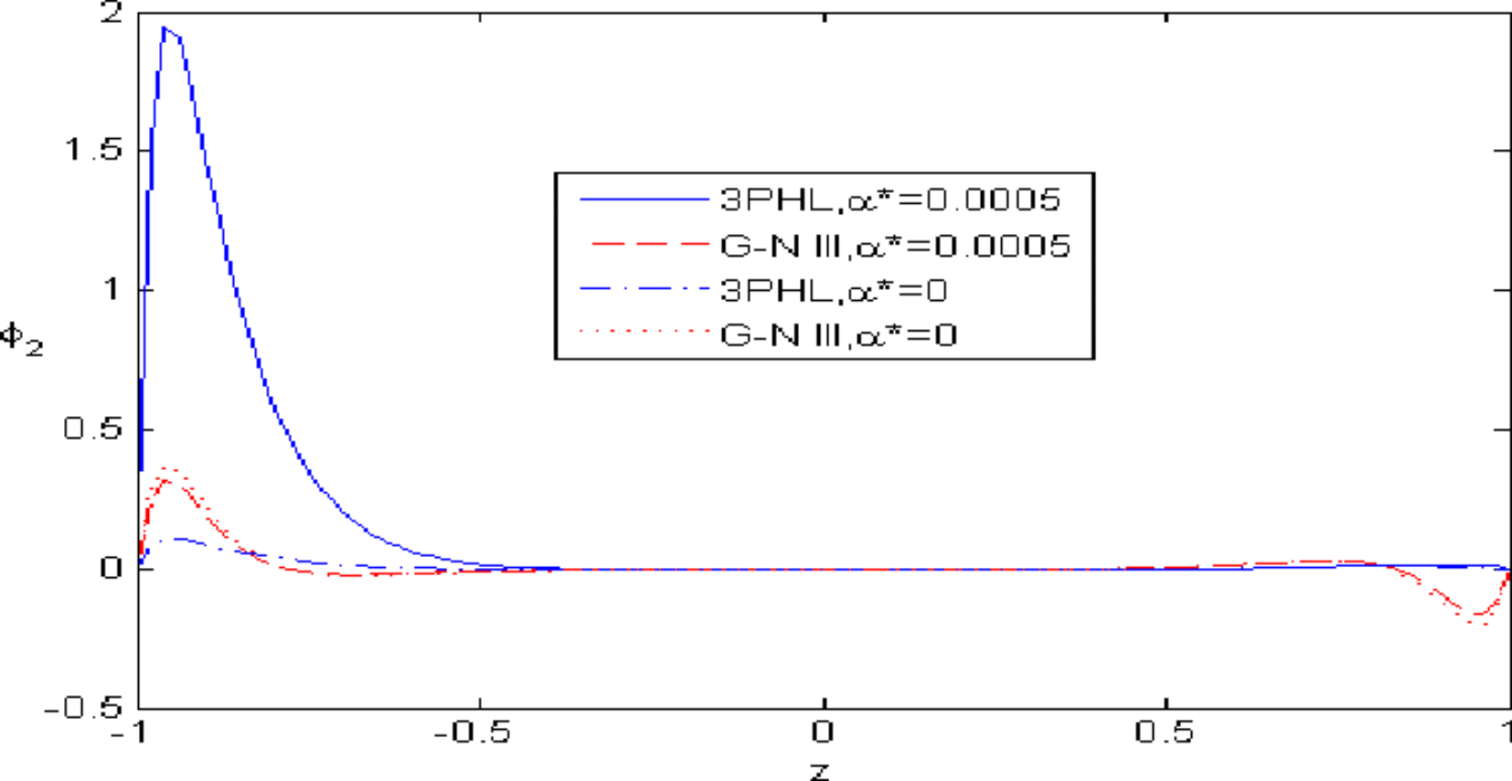
Comparing between dependent and independent temperature on $\varphi_{2} .$.

**Fig. 11:**
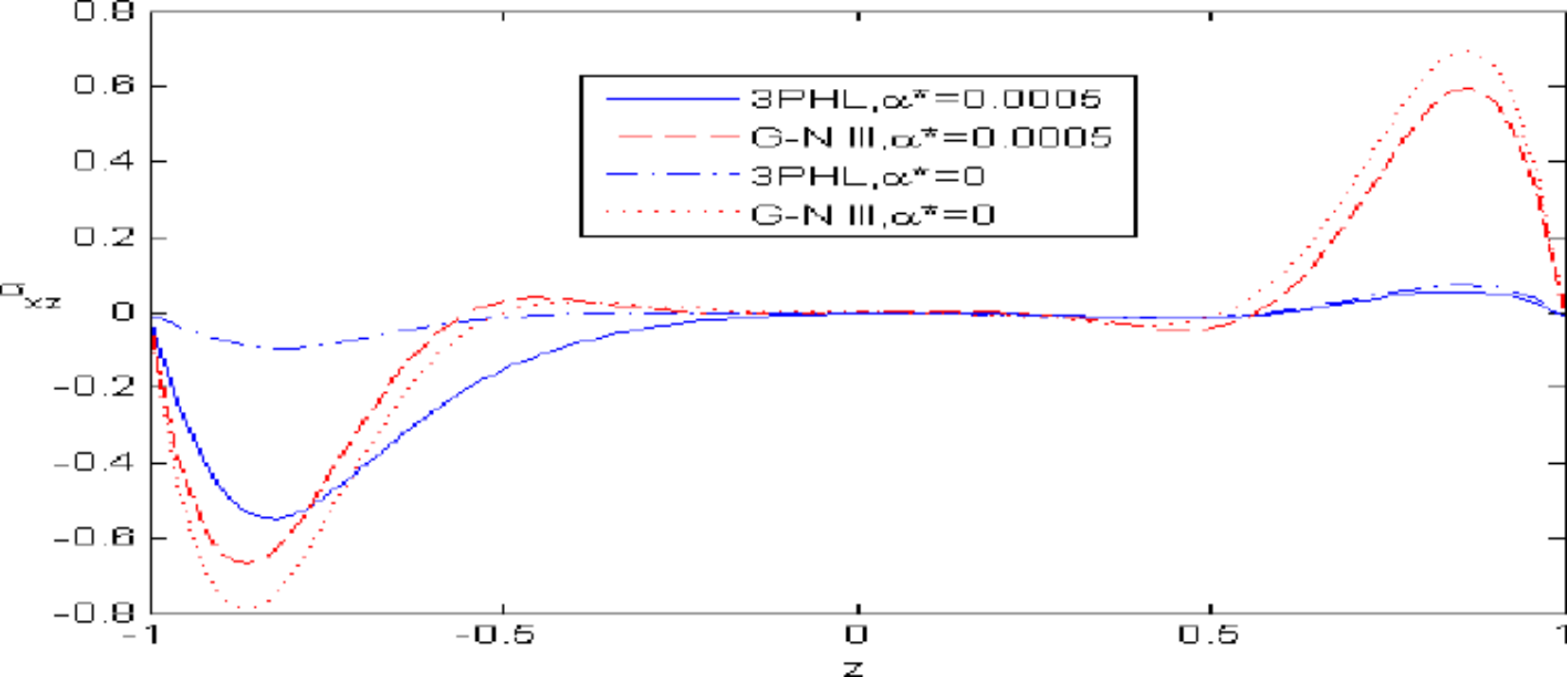
Comparing between dependent and independent temperature on $\sigma_{xz} .$.

**Fig. 12:**
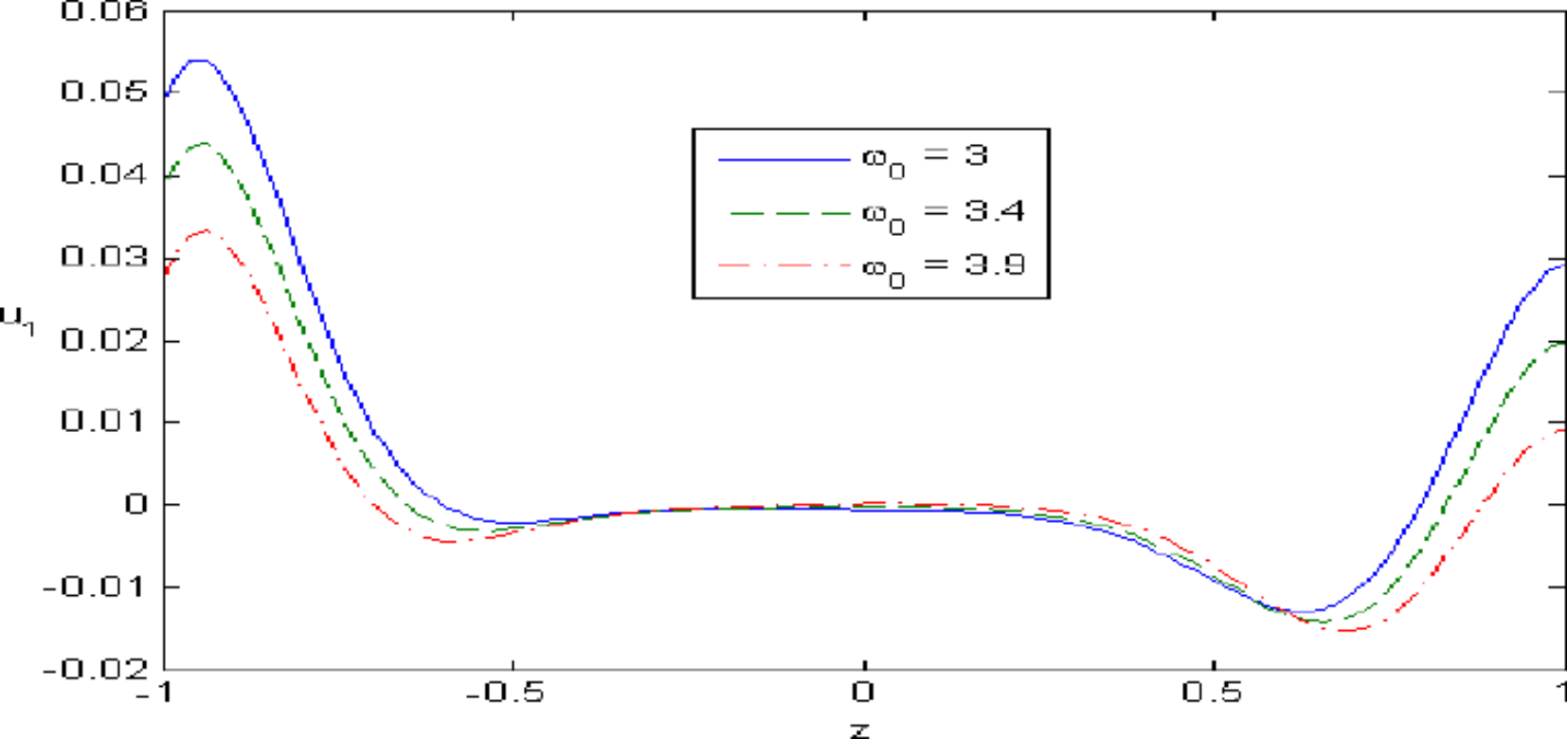
Impact of $\omega_{0}$ on $u_{1}$.

**Fig. 13:**
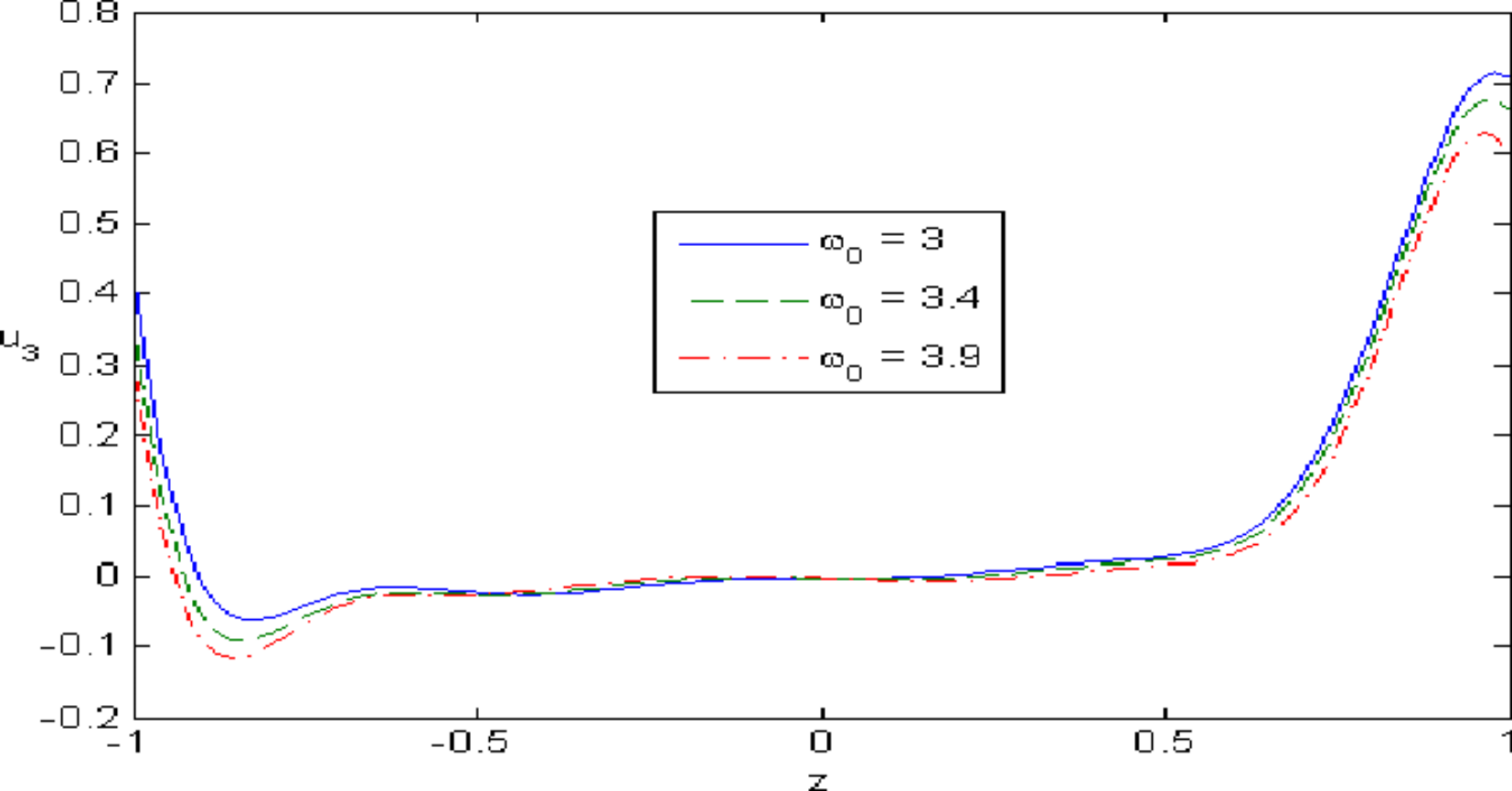
Impact of $\omega_{0}$ on $u_{3}$.

**Fig. 14:**
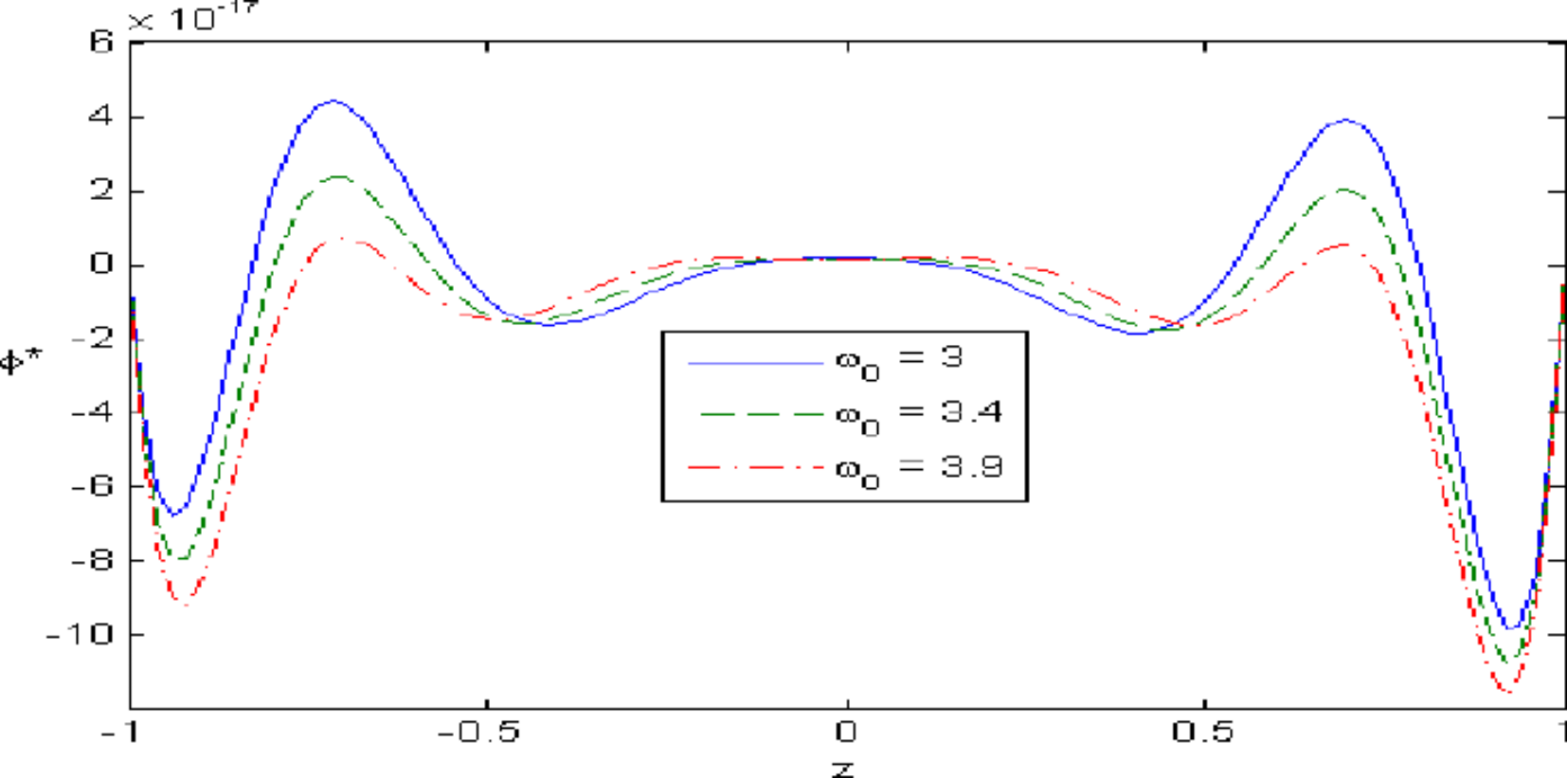
Impact of $\omega_{0}$ on $\varphi^{*}$.

**Fig. 15:**
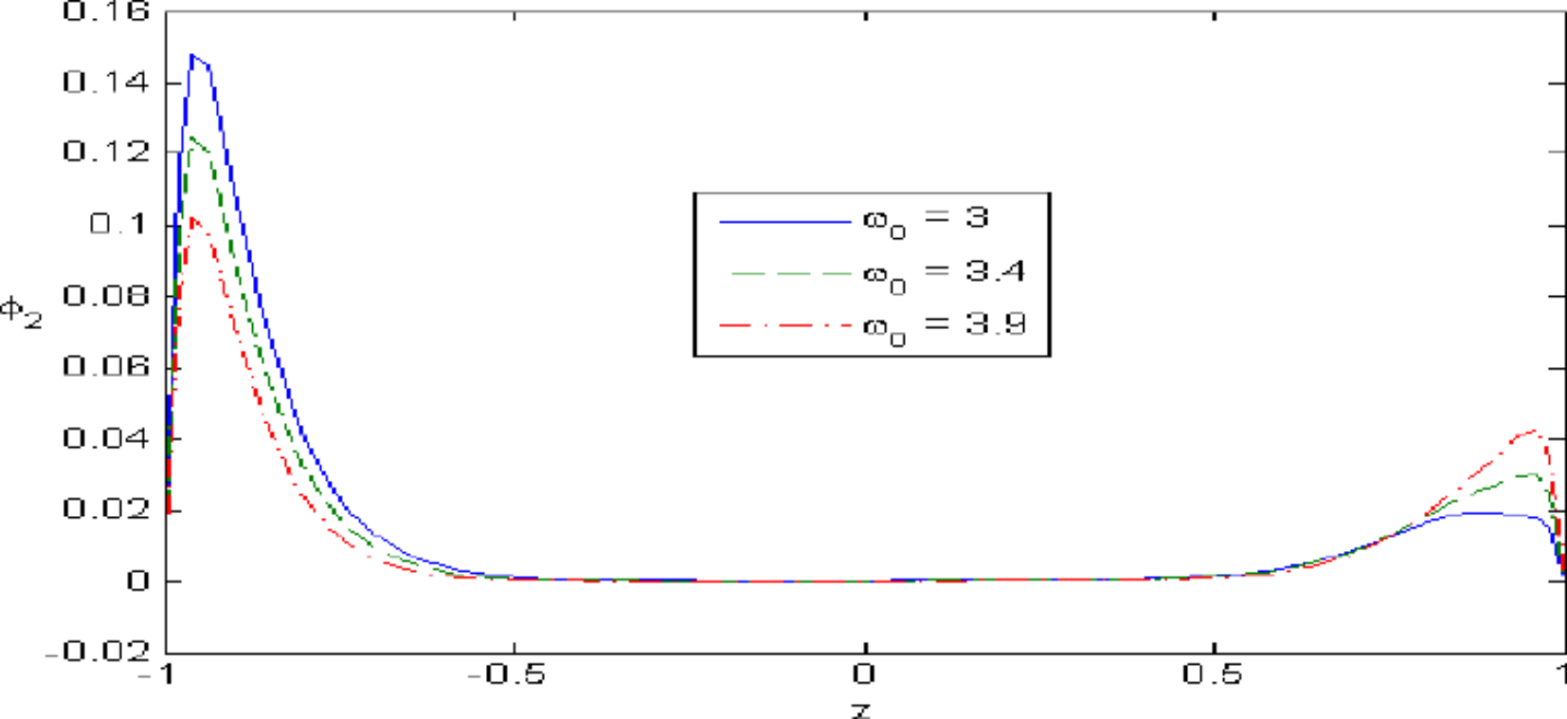
Impact of $\omega_{0}$ on $\varphi_{2}$.

**Fig. 16:**
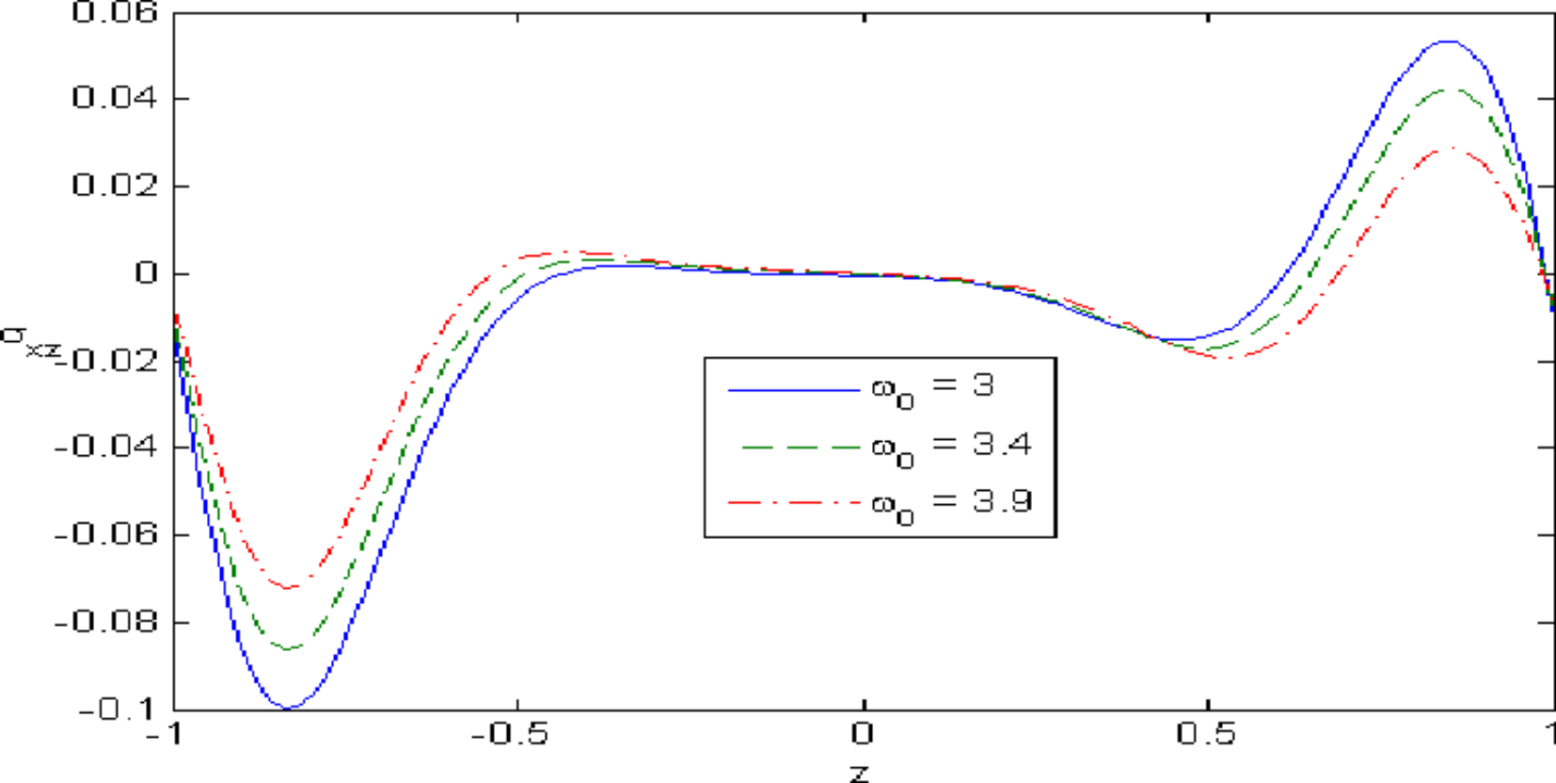
Impact of $\omega_{0}$ on $\sigma_{xz}$.

Figures 7,8,9,10,11 exhibits a comparison between the theory of (G-NIII) and the model of (3PHL) in the dependent and independent temperature. Figure 7 demonstrates the comparing between dependent and independent temperature on $u_{1} .$ In (3PHL) model, the values of $u_{1}$ in case-dependent temperature are smaller than its values in case-independent temperature, while the opposite occurs in (G-N III) theory. Figure 8 exhibits the comparing among dependent and independent temperature on $u_{2} .$ It is observed that the values of $u_{3}$ based on (G-N III) theory are greater than its values on (3PHL) model over the range $- 1\; \le \;z\; \le \;0,$ while the inverse occurs on the range $0\; \le \;z\; \le \;1.$ Figure 9 illustrates the comparing between dependent and independent temperature on $\varphi^{*} .$ It is clarified that the values of $\varphi^{*}$ based on the (G-N III) theory are smaller than its values based on the (3PHL) model at the two cases $(\alpha^{*} \;\rm{ = }\;\rm{0,}\;0.0005)$ along $z.$ Figure 10 clarifies the comparing between dependent and independent temperature on $\varphi_{2} .$ All curves start from zero and increase to a maximum value, then decrease up to zero along $z$ except the value of $\varphi_{2}$ based on (3PHL) model. Figure 11 shows the comparing between dependent and independent temperature on $\sigma_{xz} .$ All curves begin at zero and decrease to a minimum value, then increase up to zero on the range $- 1\; \le \;z\; \le \;0,$ then increase to a maximum value, then decrease up to vanish on the range $0\; \le \;z\; \le \;1.$ Figures 12,13,14,15,16 present graphical representations that illustrate and describe the changes in the aforementioned quantities with respect to $z,$ considering the presence of nonlocality in $\eta \;\rm{ = }\;0.06$ and the temperature-dependent parameter $\alpha^{*} \, = \,0.0005$ within the (3PHL) model. These figures showcase the variations for different values of the real part of the frequency $\omega_{0}$ which are listed as follows: $\omega_{0} \; = \;3,\;3.4,\;3.9.$ Figure 12 shows the impact of $\omega_{0}$ on $u_{1}$  It is noticed that the values of $\omega_{0}$ on the ranges $-1\; \le \;z\; \le \;0.45\,\,and\,\, 0.55\; \le \;z\; \le \;1.$   while the opposite occurs on the range $0\; \le \;z\; \le \;0.55.$

Figure 13 demonstrates the change of $u_{3}$ with $z$ under the influence of the $\omega_{0} .$ It is observed that the values of $u_{3}$ increase with the decrease of $\omega_{0}$ along $z.$ Figure 14 illustrates the impact of $\omega_{0}$ on $\varphi^{*} .$ It is clarified that the values of $\varphi^{*}$ increase with the increase the values of $u_{3}$  over the ranges $- \;0.4\; \le \;z\; \le \; - \,\,0.2$ and $0.2\; \le \;z\; \le \;0.4,$ while the opposite on over the ranges $- 1\; \le \;z\; \le \; - 0.4$ and $0.4\; \le \;z\; \le \;1.$ Figure 15 describes the change of micro-rotation $\varphi_{2}$ versus $z$ under the influence of $\omega_{0} .$ It is observed that the values of $\varphi_{2}$  decrease with the increase of the values of $\omega_{0}$  on the range $- \;1\; \le \;z\; \le \; - \,\,0.5$  whereas the inverse occurs on the range $0. 75\; \le \;z\; \le \; 1$. Fig. 16 shows the impact of $\omega_{0}$ on $\sigma_{xz} .$ It is noticed that the values of $\sigma_{xz}$ increase with the increase the values of $\sigma_{xz} .$ on the range $- 1\; \le \;z\; \le \; - 0.7,$ while the inverse occurs on the range $0.45\; \le \;z\; \le \;1.$** 6.**

## Conclusion

This study meticulously examines the impact of nonlocality on thermo-micro-stretch elastic materials in a temperature-dependent fluid medium, utilizing the (G-N III) and (3PHL) models. Through rigorous normal mode analysis, the investigation yields the following key insights:

1. Nonlocal effects are pivotal in thermoelastic plate deformation.
2. Clear visual comparisons between the (G-N III) and (3PHL) models, with and without nonlocality, are presented in Figs. 2,3,4,5,6.
3. Detailed comparisons of the (G-N III) and (3PHL) models under varying temperature dependencies are delineated in Figs. 7,8,9,10,11.
4. The study confirms that all boundary conditions are met by the physical quantities.
5. Significantly, the real component of the frequency parameter notably influences the physical quantities, as demonstrated in Figs. 12,13,14,15,16
6. The implications of this research extend to diverse fields such as geotechnical engineering, seismology, solid dynamics, and seismic analysis.

## Supplementary Information

Below is the link to the electronic supplementary material.

Supplementary Material 1

## Data Availability

The datasets used and/or analyzed during the current study available from the corresponding author on reasonable request.

## Abbreviations

${\varvec{u}}$: Displacement vector in micro-stretch medium
$$\alpha_{{t_{1} }} \,,\,\alpha_{{t_{2} }}$$: Linear thermal expansion coefficient, where $$\beta_{1} = \;(3\lambda \; + \;2\mu \; + k)\,\alpha_{{t_{1} }} ,$$$$\nu \; = \;(3\lambda \; + 2\mu \; + k)\,\alpha_{{t_{2} }}$$
$\lambda ,\,\mu$: Lame’s constants of classical elasticity
$$\alpha ,\;\beta ,\;\gamma$$: Elastic constants
$k$: Micropolar coupled modulus
$$\alpha_{0} ,\;\lambda_{0} ,\;\lambda_{1}$$: Stretch constants
$$J_{0}$$: Equilibrated inertia
$$J$$: Micro-inertia
$\sigma_{ij}$: Component of stress tensor
$$k^{*}$$: Additional material
$$k_{1}^{*}$$: Thermal conductivity
$$\delta_{ij}$$: Kronecker delta
$c_{E}$: Specific heat at the constant strain
$\tau_{\nu }$: Phase lag of thermal displacement gradient
$\tau_{q}$: The heat flux phase lag
$\tau_{\theta }$: The temperature gradient phase lag
$\rho$: Density for microstretch
$m_{ij}$: Component of couple stress tensor
$$e$$: Dilatation
$$\varepsilon_{ijr}$$: Alternate tensor
$\user2{\varphi }$: Micro-rotation vector
$\varphi^{*}$: Scalar micro-stretch function
$\eta$: Non-local parameter where $\eta \; = \;e_{0} \;a_{cl}$
$$e_{0}$$: Material constant
$a_{cl}$: Internal characteristic length
$\alpha^{*}$: An empirical material constant
$\lambda^{f}$: The bulk modulus
$\sigma_{ij}^{f}$: Component of stress tensor of fluid
${\varvec{u}}^{f}$: Displacement vector of fluid
$\rho^{f}$: Density of fluid
$c_{1}^{f}$: The velocity of sound of the fluid
$b$: The wave number
$\omega$: The frequency where $\omega \; = \;b\,\xi$
$i$: The complex number where $i\; = \;\sqrt { - 1} .$
